# Widespread DNA hypomethylation and differential gene expression in Turner syndrome

**DOI:** 10.1038/srep34220

**Published:** 2016-09-30

**Authors:** Christian Trolle, Morten Muhlig Nielsen, Anne Skakkebæk, Philippe Lamy, Søren Vang, Jakob Hedegaard, Iver Nordentoft, Torben Falck Ørntoft, Jakob Skou Pedersen, Claus Højbjerg Gravholt

**Affiliations:** 1 Department of Endocrinology and Internal Medicine and Medical Research Laboratories, Aarhus University Hospital, Aarhus, Denmark; 2 Department of Molecular Medicine, Aarhus University Hospital, Aarhus, Denmark; 3 Department of Clinical Genetics, Aarhus University Hospital, Aarhus, Denmark; 4 Bioinformatics Research Centre, Aarhus University, Aarhus, Denmark

## Abstract

Adults with 45,X monosomy (Turner syndrome) reflect a surviving minority since more than 99% of fetuses with 45,X monosomy die in utero. In adulthood 45,X monosomy is associated with increased morbidity and mortality, although strikingly heterogeneous with some individuals left untouched while others suffer from cardiovascular disease, autoimmune disease and infertility. The present study investigates the leukocyte DNAmethylation profile by using the 450K-Illumina Infinium assay and the leukocyte RNA-expression profile in 45,X monosomy compared with karyotypically normal female and male controls. We present results illustrating that genome wide X-chromosome RNA-expression profile, autosomal DNA-methylation profile, and the X-chromosome methylation profile clearly distinguish Turner syndrome from controls. Our results reveal genome wide hypomethylation with most differentially methylated positions showing a medium level of methylation. Contrary to previous studies, applying a single loci specific analysis at well-defined DNA loci, our results indicate that the hypomethylation extend to repetitive elements. We describe novel candidate genes that could be involved in comorbidity in TS and explain congenital urinary malformations (*PRKX*), premature ovarian failure (*KDM6A*), and aortic aneurysm formation (*ZFYVE9* and *TIMP1*).

45,X monosomy (Turner syndrome) is the only chromosome haploinsufficiency compatible with life. Nonetheless, it has detrimental effects on morbidity and mortality most strikingly affecting the 45,X fetus with less than 1% surviving pregnancy^1^. Cardiovascular mortality is increased in Turner syndrome (TS) and includes increased risk of aortic dissection and coronary artery disease^2^. Moreover, autoantibodies are present in more than half of women with TS, and close to one-fifth harbor autoantibodies against more than one organ^3^. On the other hand, the burden of comorbidity is surprisingly diverse and is not readily explained by the prevailing theory of a “simple” gene dosage imbalance following loss of sex-chromosome material^4^. Identifying causal genes of Turner syndrome comorbidity is complicated since Turner syndrome is non-heritable, making familiar-genetic analysis non-applicable, and no good animal model is available.

Both functional disomy of constitutively mono-allelic expressed genes in TS individuals with mosaic ring X-chromosomes (chrX)^5^, as well as functional monosomy of constitutively bi-allelic expressed genes^6^,^7^,^8^,^9^ have been linked to TS and morbidity, but results are equivocal^10^. Another hypothesis suggests that loss of sex-chromosome material perturbs autosomal gene transcription^11^ warranting a genome wide view on gene expression in TS. Altered autosomal gene expression as well as chrX gene expression has been shown comparing females with an iso-chromosome (46,X,iX(q)) with females with TS (45,X monosomy) on peripheral blood mononuclear cells^12^, in addition to comparisons of TS with 46,XX on amniotic fluid^13^, human fibroblast cell lines^14^, as well as induced pluripotent human cell lines^15^. Widening the scope, epigenetic alterations due to the lack of sex chromosomal material is of particular interest since epigenetics may alter gene expression. Low resolution DNA-methylation studies bring evidence of an altered methylome in 45,X fibroblast cell lines^16^,^17^,^18^ as well as in peripheral blood mononuclear cells^19^,^20^,^21^. Collectively, these studies suggest an altered global gene expression partly due to changes in the methylation profile in TS, but the literature leave a lack of studies comparing a well-characterized cohort of women with 45,X monosomy with 46,XX and 46,XY controls.

To this end, we performed leukocyte methylation profiling (450K-Illumina Infinium assay) and whole transcriptome RNA-seq in a cohort of individuals with 45,X monosomy and compared it to age-matched female and male controls. We provide evidence of global changes of the TS methylation landscape, accompanied by subtle gene expression differences with special emphasis on genes escaping X-inactivation (escape genes) and the pseudo-autosomal region.

## Results

### Widespread methylation differences and global changes in gene expression distinguish Turner syndrome from karyotypically normal males and females

**Figure 1:**
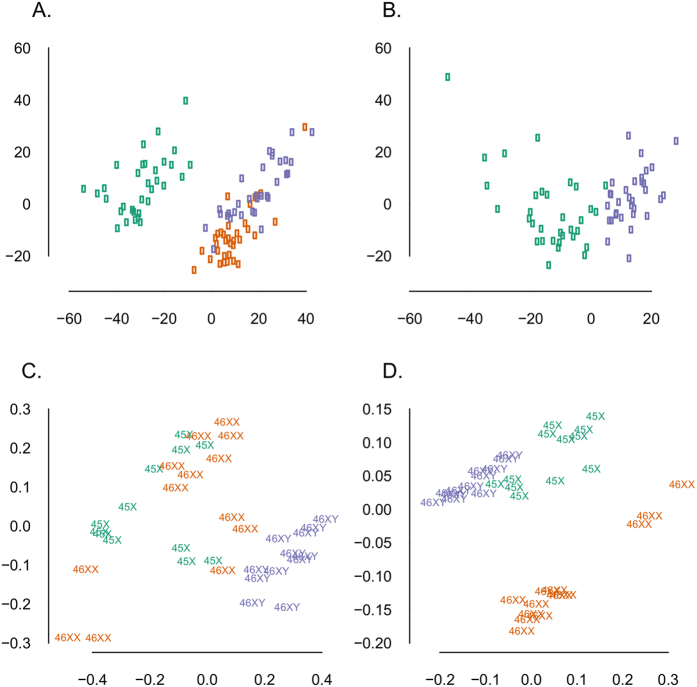
(**A,B**) Multi-dimensional scaling plots (MDSplots) based on the 2-D projection of the Euclidean distances calculated between samples using the 5000 most variable CpG positions. (**A**) Autosomal methylation data and (**B**) X-chromosomal methylation data, both illustrating clear segregation according to the TS karyoype. Note that for X-chromosomal methylation data we only compare the one X-chromosome in TS with one of males (**B**). (**C,D**) MDSplots based on the biological coefficient of variation (BCV) derived from the expression data. (**C**) Autosomal expression data with no clear clustering. (**D**) X-chromosomal expression data showing clustering according to karyotype. Green = Turner syndrome; Purple = Male controls; Orange = Female Controls.

**Figure 2:**
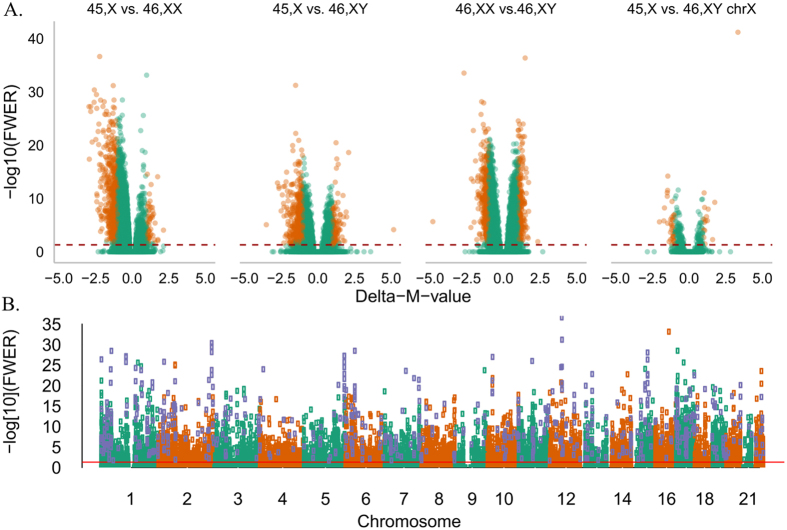
(**A**) Volcano plots of – log 10 (Family Wise Error rate = Bonferroni) against delta-M-values representing the methylation differences, adjusted for age and the relative cell proportions. Orange dots are differentially methylated positions (DMPs) exceeding the threshold (FWER < 0.05 and absolute (delta-M-value) > 1. (**B**) Manhattan plot of DMPs from the 45,X vs. 46,XX comparison. Red line is FWER = 0.05. DMPs with an overall significant FWER < 0.05 and absolute (delta-M-value) > 1 are purple. The overall hypomethylation seen in Turner syndrome (**A**) and the genome wide nature of this pattern is evident (**B**) with hypergeometric test revealing enrichment of chromosome 1, 11, 17 and 22 and the proximal promoter.

Evidence suggests that sex chromosomes regulate gene expression throughout the genome with an influence on 3% of autosomal genes^11^. We speculated that this regulation was traceable in the TS methylome and in the transcriptome of the chrX. We used the 450K-Illumina Infinium assay to study DNA-methylation in 33 women with TS and in an equal sized cohort of age-matched female and male controls, in addition to RNA-seq expression profiling in 12 TS, 13 46,XX females, and 12 46,XY males. Supporting our hypothesis, multi-dimensional scaling plots (MDSplot) and hierarchical cluster analysis, based on both methylation data and expression data, revealed a distinct TS cluster (Fig. 1, Supplemental Figs 1 and 2). We compared methylation on the one active chrX of TS with the one active chrX of 46,XY males, revealing 200 differentially methylated positions (DMPs), 43 with a delta-Beta > 0.1. Extending the model to the autosomes and comparing all three karyotypes, we observed a varying number of DMPs between the groups. The most pronounced differences were in the TS vs. 46,XX comparison, with predominantly proximal promoter hypomethylation. Intermediate differences were found in the 46,XX vs. 46, XY comparison. The most comparable profiles were in the TS vs 46,XY comparison. In summary, the three comparisons revealed 635, 787, and 760 DMPs (absolute delta-M-value > 1), respectively (Fig. 2A,B). Applying a cut-off of delta-Beta > 0.1, 469 (TS vs. 46,XX), 78 (46,XX vs. 46,XY), and 293 (TS vs. 46,XY) reached significance.

**Figure 3:**
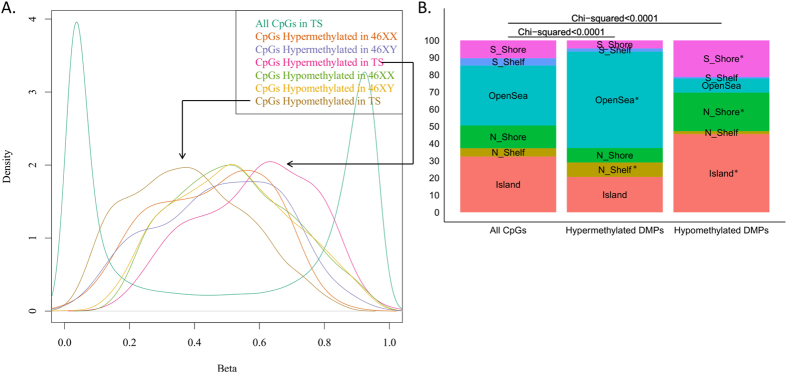
(**A**) Distribution of methylation values in Turner syndrome showing the normal biphasic distribution of all CpGs (in green) with a spike close to zero and close to one. In comparison, methylation values corresponding to hypomethylated differentially methylated positions (DMPs) (purple) and hypermethylated DMPs (orange) in Turner syndrome showed nearly overlapping curves, approximately normally distributed, with median values at 0.37 and 0.62, respectively. Hence, most affected values show a medium level of methylation. (**B**) CpG island status of hypermethylated and hypomethylated DMPs were significantly different compared to all CpGs on the array. Enriched location are marked by “P < 0.0001” in plot.

We subsequently explored the distribution of methylation values in the TS cohort. Methylation values corresponding to significantly hypomethylated DMPs and hypermethylated DMPs showed nearly overlapping curves and both were approximately normally distributed, with median values at 0.37 and 0.62, respectively (Fig. 3A). Hence, most affected DMPs show a medium level of methylation. CpG island (CGI) status of hypermethylated and hypomethylated DMPs were significantly different compared to all CpGs on the array (Fig. 3B). Hypomethylated DMPs were enriched with respect to Islands and their shores, while hypermethylated DMPs were enriched with respect to open-sea and shelfs (Fig. 3B). In addition, the hypergeometric test revealed DMP enrichment of chromosome 1, 11, 17 and 22 and of the proximal promoter (Fig. 2B).

**Figure 4:**
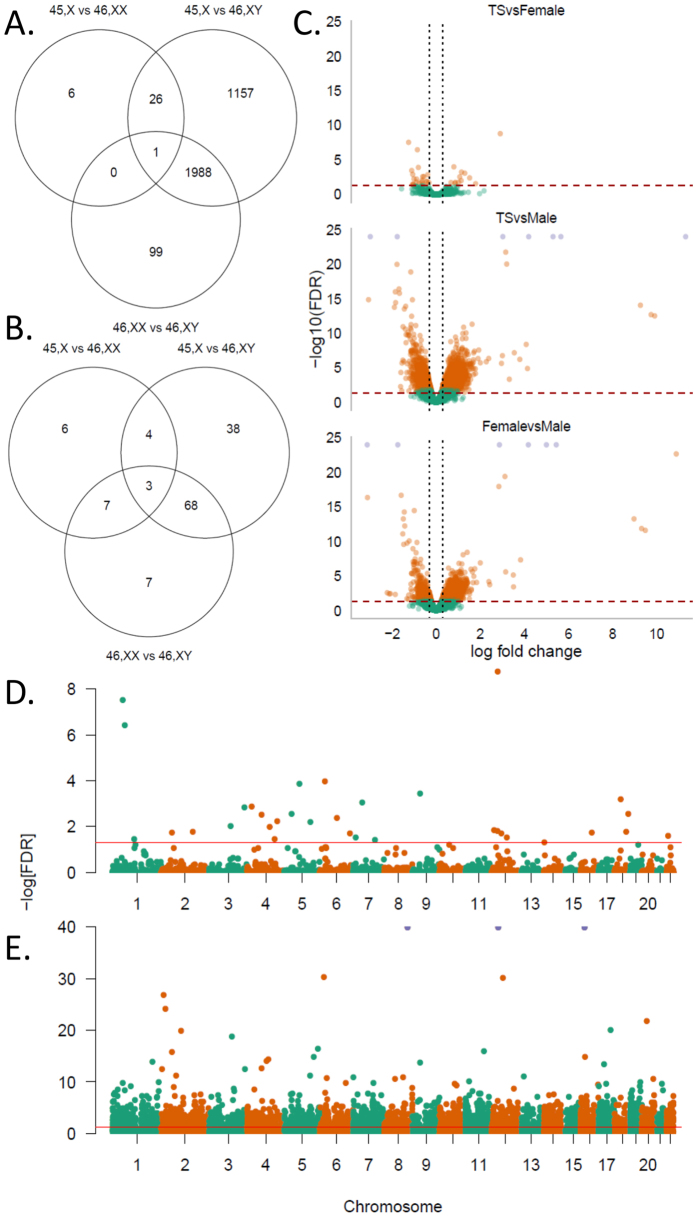
Plots are based on RNA-seq data and illustrate the global changes in gene expression affecting individuals with Turner syndrome. (**A**) Venn diagram of autosomal genes. and (**B**) of sex-chromosomal genes. (**C**) Volcano plot of autosomal genes illustrating that differential expressed genes are both up and down-regulated. Orange dots are genes with an absolute log fold change ≥ 0.3 and FDR < 0.05. (**D,E**) Manhattan plot of autosomal genes from the Turner syndrome vs. females and Turner syndrome vs. males comparison with differential expression affecting close to all autosomes. Level of significance is set at Overall FDR and individual FDR < 0.05 and absolute log fold change ≥ 0.3.

After having described profound hypomethylation at the level of single CpGs, we proceeded to explore the differences in methylation between the three karyotypes at a regional level. By using adjacent site clustering, we identified 15 chrX differentially methylated regions (DMRs) and 400 autosomal DMRs (Supplemental Fig. 3). To assess if these differences in methylation, or a lack of transcription factor expression due to the reduced chrX dosage, have an impact on chrX or autosomal gene expression we analyzed gene expression differences between the groups. Intergroup comparisons were done on the chrX with TS vs. 46,XX revealing 20 (2 up-regulated and 18 down-regulated), TS vs 46,XY: 113 (52 up-regulated and 61 down-regulated), and 46,XX with 46,XY 85 (48 up-regulated and 37 down-regulated) differentially expressed genes (FDR < 0.05; absolute log fold change ≥ 0.3, corresponding to a fold change above ≥ 1.2, a cut-off used previously in a TS study^12^). These differences extended to the autosomes with 33 (TS vs. 46,XX: 12 up-regulated and 21 down-regulated), 3,172 (TS vs. 46,XY: 1,836 up-regulated and 1,336 down-regulated) & 2,088 (46,XX vs. 46,XY: 1,382 up-regulated and 706 down-regulated) differentially expressed genes (Fig. 4) with 26 genes differentially expressed in both TS contrasts (Supplemental Table 1).

The relationship between DNAm and expression is complex and amongst others depends on whether the CGI is located at a promoter or is a so-called orphan CGI, that is within or between characterized transcription units^22^. We explored the correlation between DNAm and gene expression at DMPs within orphan CGIs (corresponding to 53 genes) and at proximal promoters (34 genes). At five autosomal genes, but no Xchr genes, we found a moderate to strong correlation between the methylation status at orphan CGIs and gene expression (Supplemental Table 2 and Supplemental Fig. 4).

In summary, we observe that genome-wide hypomethylation as well as differential gene expression not only affect the chrX but also the autosomes. We have demonstrated how methylation differences and differences in chrX expression clearly distinguish 45,X monosomy from controls.

### Methylation at repetitive elements

Previous studies have explored the DNAm level at repetitive elements, such as long interspersed elements (LINEs), indicating a correlation between the methylation level and the genome size, with hypermethylation of LINEs in smaller genomes (45,X) and hypomethylation in larger genomes (47,XXY)^20^. In parallel, recent extensions of the 450K-Illumina Infinium assay^23^ allowed us to assess the methylation level at autosomal repeats comparing TS with 46,XX controls. We clustered probes on the same chromosome and separated by less than 500 base pairs defining in total 1404 regions in addition to the 19,468 Illumina probes hybridizing to repetitive elements. 29 DMPs (27 hypomethylated and 2 hypermethylated) and 8 hypomethylated regions reached a FWER < 0.05 and a delta-Beta value above 0.1 (Supplemental Fig. 5). Secondly, we assessed the number of DMPs in repetitive elements compared with the number of DMPs in the remaining part of the genome and no enrichment was found with respect to repetitive elements.

### Sex chromosomal escape genes and pseudo-autosomal genes are prone to differential expression in Turner syndrome

Mono-allelic expression of normally bi-allelic expressed sex chromosomal genes may in part explain the TS phenotype. Such candidate genes are X-Y gene pairs, genes of the pseudo-autosomal regions, as well as escape genes. We hypothesized that loss of sex-chromosome material in TS will change the transcription and translation of chrX genes depending on whether the genes are subject to X-chromosome inactivation (XCI). In addition, we speculated that dosage compensation of TS relative to controls might occur at multicopy or ampliconic X-linked genes, in contrast to single-copy genes, as a result of buffering or feedback mechanisms^24^,^25^. Consequently, we expected none or few differentially expressed multicopy/ampliconic genes.

**Figure 5:**
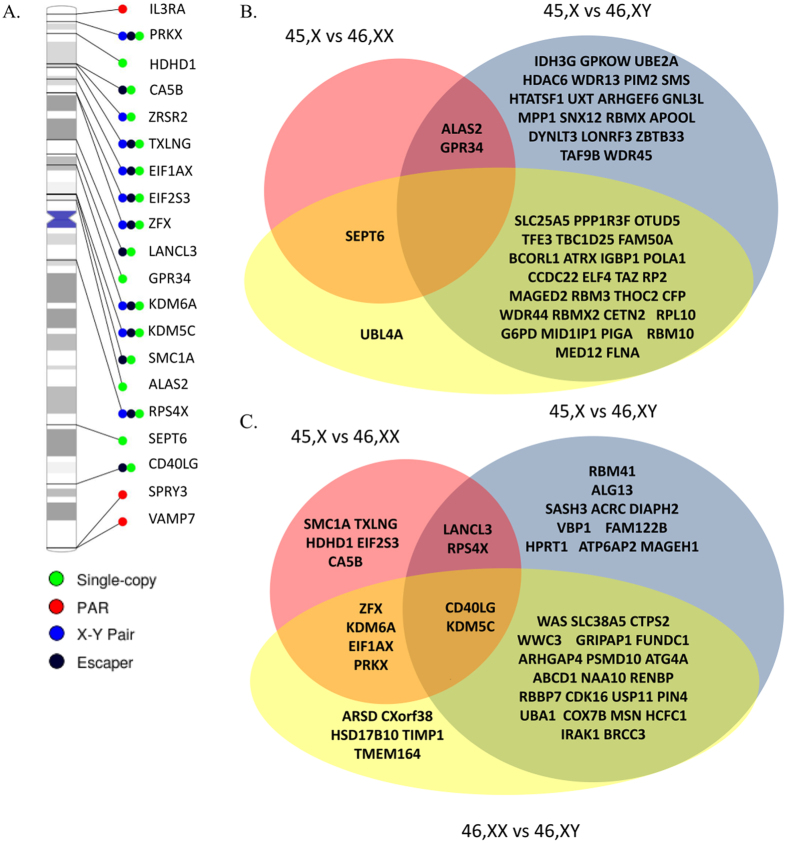
(**A**) Differentially expressed X-chromosomal genes from the 45,X vs 46,XX comparison plotted corresponding to their location on the X-chromosome and with color coding according to status: Escape gene (black), Pseudo-autosomal (green). Single copy (red), and X-Y pairs (blue). Transparent red regions on the ideogram are the pseudo-autosomal region 1 and 2. Names are HGNC gene symbols. Plot illustrates that all differentially expressed genes were single copy genes or genes normally bi-allelic expressed with no multi-copy or ampliconic genes being differentially expressed. (**B,C**) Venn diagram listing differentially expressed X-chromosomal genes with Overall FDR < 0.05 and individual FDR < 0.05 and annotated as either inactivated (**B**) or as escape gene (**C**).

We annotated 51 escape genes according to Bellott *et al*.^11^ and found *LANCL3*, *RPS4X* and *JPX* differentially expressed in both TS contrasts (Supplemental Table 3 and Fig. 5). *RPS4X* (Supplemental Fig. 6) has been associated with TS previously^6^,^26^, and *JPX* reported to escape XCI and activate *XIST* on the inactive X. Of genes being classified as inactivated^11^, *ALAS2* and *GPR34* were common to both the TS contrasts (Supplemental Table 3 and Supplemental Fig. 7) with *ALAS2* expression being increased two-fold in TS. It must be kept in mind that for several X chromosomal genes a Y homolog exist (Supplemental Table 4) possibly explaining an apparent sex-biased expression. We found differential expression of five of these genes: *EIF1AX, ZFX, KDM6A*, and *KDM5C*. Considering their Y homologs, we investigated the expression of the respective X-Y pairs. This revealed an increased expression level of *EIF1AX, ZFX, KDM6A* and *KDM5C* among males (46,XY), with no change in the expression level in TS, and 46,XX (Supplemental Fig. 8). Interestingly four of the escape genes (*KDM6A, STS, UBA1*, and *USP9X*) were as well differentially methylated (Supplemental Fig. 9 and Table 1).

Pseudo-autosomal genes are, like escape genes, bi-allelic expressed. Hence, loss of sex chromosomal material corresponding to these regions may explain phenotypic features of TS, as exemplified by SHOX haploinsufficiency involved in TS low stature^2^. Still, due to multi-mapping issues, these genes are often lost during mapping of short read sequence data. We therefore separately mapped reads only to the pseudo-autosomal region. This resulted in expression estimates of 26 genes, of which three (*VAMP7, SPRY3, IL3RA*), five (*P2RY8, PLCXD1, WASH6P, CSF2RA*, *CD99*), and two (*CD99, IL9R*) genes were differentially expressed in the TS vs. 46,XX, TS vs. 46,XY, and 46,XX vs 46,XY, respectively (Supplemental Figs 10 and 11). Three of these genes code for proteins involved in immune responses (*CD99*: T-cell adhesion, *IL3RA*: Receptor for Interleukin-3, *CSF2RA*: Colony stimulating factor 2 receptor) and thus may play a role in the predisposition for autoimmune disease in TS and, contribute concurrently to embryonic lethality. Also, *CSF2RA* was previously suggested as a candidate gene explaining the early lethality of TS embryos^27^.

To illustrate if differentially expressed genes were multi-copy, ampliconic, or single copy genes, we adapted the annotation by *Bellott el al*.^11^ and *Mueller et al.*^28^. As illustrated in (Fig. 5A) all differentially expressed genes were single copy genes or genes normally bi-allelic expressed, and thus no gene, thought to be a multicopy gene or an ampliconic gene, were differentially expressed.

In summary, analysis of the sex chromosomes reveals differential expression of escape genes and pseudo-autosomal genes, but also genes normally subjected to XCI. In addition, the results support our hypothesis that buffering mechanisms exist for multicopy/ampliconic genes. Lastly, we find three candidate genes for the autoimmune predilection and one for the high in utero lethality.

### Increased differential methylation and differential expression of genes associated with co-morbidities in Turner syndrome

A high prevalence of autoimmune disease and cardiovascular comorbidity characterize TS, however without obvious candidate genes. To this end, we annotated DMPs to genes and performed gene set enrichment analysis. Running the gene-centric modular analysis, the genetic association database revealed enrichment for terms related to the metabolic syndrome, type-2 diabetes, and sensorineural hearing loss as well as liver disease and others (Supplemental Table 5). This finding goes well in hand with these comorbidities being highly prevalent in TS^29^. For example, TS patients have a 4-fold overrepresentation of type 2 diabetes, and around 50% suffer from sensorineural hearing loss. In addition, we ran DAVID on the 33 differentially expressed autosomal genes from the TS vs. 46,XX comparison. We found that the genes segregated and were connected through Gene Ontology terms with the potential to affect cell functioning (Supplemental Table 6). Still, the analyses of DMPs are restricted by the annotation of Illumina probes to single genes. We thus expanded the analysis from the single gene/DMP approach and used GREAT^30^ to relate DMRs to possible candidate genes. The 15 chrX DMRs (Supplemental Fig. 3) related to seventeen genes involved in mental retardation, histone acetylation, and histone deacetylation. Interestingly, one DMR was annotated to the *TIMP1* gene, previously suggested to participate in aortic aneurysm formation^31^,^32^, which is a highly prevalent condition in TS. The 400 differentially expressed autosomal regions (DMRs) revealed GO-terms implemented in chromosome organization, embryonic development, and morphogenesis (Supplemental Table 7).

In summary, our results are associated with genes involved in diseases commonly affecting TS and with Gene Ontology terms related to chromosome organization, embryonic development, and morphogenesis.

### Genes possibly implemented in fetal development and chromatin loss show differential exon-usage

Gene-level count-based methods for assessing differentially expression may not accurately represent the true difference in gene expression since they do not detect differences in the expression of transcript isoforms. Changes in isoform transcript expression could be prevalent in TS where the genome-wide DNA hypomethylation might reactivate cryptic transcription start sites. In addition, it is possible that differentially expressed splice factors could contribute to the TS phenotype. To this end, we evaluated differential exon usage with DEXSeq and report results from the TS vs 46,XX comparison. Surprisingly, only one chrX gene (*VSIG4*, FDR < 0.05 and absolute log fold change ≥ 0.3) showed differential exon-usage. Adjusting for the overall expression level within the studied groups (Supplemental Fig. 12) did not change the result. *VSIG4* is likely to be involved in general fetal development and pulmonary function with highest expression in placenta, lung, adrenal gland, heart, and liver^33^,^34^. *VSIG4* expression in macrophages suggests that it may be an inhibitory ligand maintaining T-cell unresponsiveness in healthy tissues^35^. Applying our analysis of differential exon-usage to the autosomes, eight protein coding (*HAL, ACSS3, STIL, TUBGCP6, SPAG16, BUB1B, RGS12, SNTB2*) and two non-coding RNAs (*AC159540.1, RP11-664D1.1*) genes were differentially spliced (Supplemental Table 8). *BUB1B* (Supplemental Fig. 13) might play a role in Mosaic Variegated Aneuploidy syndrome. We speculate that it may also play a role in the loss of chrX material in TS, analogous to the *FMR1* gene, which may predispose to chromosomal loss during mitosis^9^. In summary, 12 genes showed differential exon-usage, two of which are of evident interest in light of TS comorbidities.

### Comparison with previous aneuploidy studies utilizing the 27K Illumina array

The 27K Illumina array is to a vast extend contained within the 450K-Illumina Infinium assay enabling us to compare our results with that of Sharma *et al*.^21^, who studied the DNAm pattern in 45,X, 46,XX, 47XXY, and 46XY using the 27K array. All but three of the significant DMPs from our study - mapping to the 27K array - were also found to be differentially methylated in the study by Sharma *et al*.

## Discussion

This study illustrates that 45,X monosomy is associated with genome wide DNA hypomethylation in both cis and trans, and to a lesser extent areas of hypermethylation. We provide evidence for an altered expression of both autosomal and sex chromosomal genes and point to several candidate genes not hitherto described in TS. With more than 99% of fetuses with 45,X monosomy dying in utero, we suggest viewing adults with TS as a surviving minority resulting from a process of strong selection. In addition, we provide evidence suggesting several candidate genes possibly involved in the diverse clinical presentation of 45,X monosomy.

### A unique Turner syndrome expression profile and DNA-methylation profile

Apart from contributing a unique well-annotated dataset with expression and methylation measures, our study provides evidence of a unique TS gene regulation as assessed by methylation and RNA-seq expression profiling. Based on these profiles, our cohort segregated into three groups according to karyotype (Fig. 1A,C). This indicates global specific changes in the methylation-profile in relation to the karyotype and thus confirms and extends a previous small, low-resolution methylation study using Illumina’s 27K assay^21^. The widespread hypomethylation of proximal promotors in our study makes it likely that the findings have regulatory impact on gene transcription and hence suggest a possible link between the differential methylation and the differential expression seen in our study.

With respect to the chrX, our results suggest, that the chrX of TS has a distinct methylation profile when compared to the chrX of 46,XY males (Fig. 1B,D). A novel finding, since previous studies have generated methylation-profiles of the inactive chrX of 46,XX females by subtracting the chrX methylation-profile of TS from that of 46,XX women. This method was based on the assumption that the active chrX of TS was comparable to that of 46,XX^19^, which thus probably is not the case.

### Methylation status at repetitive elements

DNAm of repetitive elements contributes to genomic stability in preventing transposition and illegitimate recombination, in addition to silencing of cryptic start sites, and preventing transcriptional interference from strong promoters^36^. The hypomethylation seen at repetitive elements in our study is different to previous studies^20^, which used a single loci specific analysis at well-defined DNA loci, while the methodology that we have used, is based on a global approach. This discrepancy is possibly explained by the differing methods, the larger sample size, or the exclusion of mosaic karyotypes in our study. To accommodate problems with the loci specificity of the 450K-Illumina Infinium assay at repetitive elements we adapted the annotation from Price *et al*.^23^, defining repetitive elements as non-cross reacting probes with the entire probe (50 base pairs) in repetitive DNA. Interestingly, taking into account all probes, we did not see enrichment for repetitive elements, and as such, DMPs seem to be randomly distributed also affecting repetitive elements.

### Candidate genes of Turner syndrome co-morbidity on the X-chromosome

Previous evidence suggests that the sex chromosomes contribute to a major genetic difference when comparing males and females, opposed to intra-sex comparisons^37^. Such differences may potentially explain the sexual dimorphism in health and disease as exemplified by the increased female incidence of autoimmune disease and the male preponderance of cardiovascular disease. 45,X monosomy, with its high prevalence of both diseases with a female and a male preponderance, provides an extreme phenotype to study the impact of sex-chromosome haploinsufficiency.

In our study, the sex-chromosome analysis provided the largest set of differential expressed genes (Fig. 5). Furthermore, the inclusion of both sexes as controls illustrates the unique TS expression profile. We hypothesized that these TS genes would be escape genes and/or genes of the pseudo-autosomal region. *RPS4X, JPX*, and *LANCL3* represent three such escape genes. *RPS4X* encodes ribosomal protein S4, previously suggested to play a role in TS, as individuals with deletions of the *RPS4X* homolog part of the Yp (*RPS4Y1*) display somatic features similar to TS^6^,^38^, although not consistently in human^39^ nor animal studies^26^. However, these studies have focused on the apparent phenotypic traits of TS e.g. short stature, infertility, gonadal dysgenesis, and congenital malformation. We speculate, that *RPS4X* relates to less apparent TS stigmata, and find it interesting that *RPS4X* continue to surface in RNA-seq TS studies^14^,^15^. *JPX*, parallel to *TSIX*, is a RNA-switch activating *XIST* on the inactive chrX. Correspondingly, we found *JPX* to be downregulated in TS. This finding fits well with the biological function of *JPX*. Likewise, *XIST* itself was differentially expressed in three^14^,^15^ of the five genome-wide expression studies, including our own. In addition, we found differential methylation of four escape genes (*KDM6A, UBA1, STS, USP9X*; Supplemental Fig. 9 and Table 1). Interestingly, *KDM6A* (taking into account its Y-homolog; Supplemental Fig. 8D) showed differential expression while *UBA1*, *USP9X* and *STS* did not. This paucity of differential methylation within the pseudo-autosomal region may be explained by the sparse coverage of the pseudo-autosomal region by the 450K Illumina array and the fact that many probes are filtered out due to cross-hybridization. In addition to escape genes, ten pseudo-autosomal genes were differentially expressed (Supplemental Figs 10 and 11) with three coding for proteins involved in immune responses possibly playing a role in the autoimmune co-morbidity in TS.

X-chromosomal genes, differentially expressed in all three comparisons (*CD40LG* and *KDM5C*), were also of interest. Previous papers links *CD40LG* to the Hyper-IgM syndrome with secondarily increased prevalence of autoimmune diseases as sero-negative arthritis, hypothyroidism, hepatitis, and inflammatory bowel disease^40^. All these conditions are seen with increased frequency in TS, and may thus play a role in TS autoimmunity. *KDM5C* participates in transcriptional repression of neuronal genes by recruiting histone deacetylases and RE-1-silencing transcription factor (REST) complex at neuron-restrictive silencer elements, being important for the transcriptional repression of neuronal genes. We speculate that *KDM5C* may play a role in the distinct neuro-cognitive profile of TS. We found several differential expressed genes previously reported in TS research (*EIF1AX*^15^, *ZFX*^9^,^14^,^41^,^42^, *PRKX*^14^, and *RPS4X*^6^,^10^,^14^,^15^) with some mentioned as sex biased genes (*KDM5C*, *KDM6A*, *EIF1AX*, *ZFX*, and *PRKX*)^43^. *PRKX* is suggested to regulate epithelial morphogenesis during kidney development^44^. This is an interesting finding since women with TS have a 8-fold increased relative risk of congenital urinary malformation and presence of horse-shoe kidney^29^. Likewise, *KDM6A*s importance for reestablishment of pluripotency and germ cell development^45^ makes it a potential candidate gene in the ovarian failure in TS. Belonging to the Homeobox gene-family, *KDM6A* help to direct tissue differentiation in cardiac cell as well as myogenesis^45^ with the potential to be involved in the abnormal cardio-vascular development in TS. Interestingly, KDM6A and overlapping congenital cardiovascular abnormalities are a common denominator in TS and Kabuki syndrome, where this gene is mutated^45^,^46^,^47^,^48^.

Studying X-Y homologs bias can be introduced when comparing gene expression between groups with differing number of sex chromosomes, because these gene pairs are counted as separate genes although evidence suggests they may serve the same function. However, taking this into account, the overall expression pattern only changed in the 46,XY group (Supplemental Fig. 8) consistent with lack of Y-chromosome material in the two other groups.

In keeping with our hypothesis none of the differentially expressed genes from the TS vs. 46,XX contrast were multicopy or ampliconic genes but rather genes normally bi-allelic expressed or single copy genes (Fig. 1). We posit that dosage compensatory mechanisms (e.g. increased transcription or extended RNA half-life^24^) may be more adapted to dampen effects of changes in copy number of these genes in contradiction to single copy or bi-allelic genes.

### Candidate genes of Turner syndrome comorbidity on the autosomes

Studies of sex-biased gene expression indicate that X-Y gene pairs regulate expression across the genome in line with enrichment of X-Y gene pairs for regulators of transcription, translation and protein stability^11^. Correspondingly, loss of sex-chromosome material could carry an impact on autosomal gene expression resulting in a divergence in gene expression in TS. We found 33 out of 10,174 autosomal genes to be differentially expressed when comparing TS with 46,XX indicating that loss of sex-chromosome material alters autosomal gene expression. Exploring the individual genes, several of them were related to morbidity in TS e.g. aortic aneurysm (*ZFYVE9*), obesity (*CNR1*), melanocytic nevi (*DOCK7*)^49^, and the insulin-like-growth factor system (*IGFBP3*), which is known to be perturbed in TS^50^. Studies on the pathogenesis behind aortic aneurysms in Marfan syndrome and individuals with bicuspid aortic valves indicate a role of SMAD2 in aneurysm development^2^. Therefore, we find it intriguing that *ZFYVE9*, which recruits SMAD2 to the TGF-β receptor, was differentially expressed and therefore could contribute to aortic dilatation in TS.

The growth hormone-IGF-1-IGF-binding-protein system is perturbed in TS with lower levels of IGF-1 and increased IGFBP-3 proteolysis^50^. We propose that the up-regulation of IGFBP3-mRNA in our study is a consequence of the increased IGFBP-3 proteolysis.

Not only autosomal RNA expression but also the autosomal methylation profile was perturbed in TS with pathway analysis relating the affected DMPs to type-2 diabetes, the metabolic syndrome, hypertension, obesity, short stature and impaired hearing (Supplemental Table 5); and DMRs to GO-terms predominantly involved in embryonic development or morphogenesis (Supplemental Table 7). This is interesting in light of the high prevalence of congenital cardiovascular and renal abnormalities in TS. Yet, this widespread hypomethylation did not seem to activate cryptic transcription start sites with the exon-usage being comparable with the exception of a few interesting genes e.g. *VSIG4* and *BUB1B.* The latter is of interest as it is a cause of aneuploidies. We speculate that the differential exon-usage seen in TS may play a role in the loss of chrX material in TS. Still, we recognize that the differential-exon-usage of *BUB1B* may also be a consequence of the chrX monosomy itself. Further studies focusing on this gene in other sex-chromosome aneuploidies are required to shed light on the issue.

The epigenetic differences presented here are more extensive than expected from studies on comorbidities such as diabetes or rheumatoid arthritis. Hence, it is unlikely that the higher incidence of co-morbidity in TS drives the observed epigenetic differences. In addition, the recruited female controls were healthy controls when originally matched with our TS cohort for our prospective study on aortic dilatation. When recruiting the same cohort for the present study (on average 6 years later) some controls have developed comorbidities such as hypertension and reflux esophagitis. This is a confounder but if anything adds to the variability in the epigenetic data, making it more difficult to prove a difference between cases and controls and these comorbidities does not explain the widespread epigenetic differences in our study. Using animal models would be attractive, but in the case of TS, no optimal models exist. For example, the so called TS mouse model is fertile, and striking differences exist between human and mouse regarding the identity and number of escape genes^51^. Lastly, our methylation-based and expression-based pathway analysis were complementary rather than overlapping. This finding probably attributes to the fact that expression data was only available on a subgroup. Furthermore, the DNAm is also a mark of cell differentiation and to a wide extent a static pattern marking the differentiation of the tissue studied^52^. Ultimately, evidence suggests DNAm as an epigenetic memory with e.g. hypomethylation at enhancers active during embryonic development but dormant in adult tissues^53^, thus not necessarily illustrated by the prevailing RNA expression data.

### Distribution of DNA-methylation

Overall chromosome 1, 11, 17 and 22 and the proximal promoter were enriched for DMPs. We found CGI status of hypermethylated and hypomethylated DMPs to be significantly different compared to all CpGs on the array. Hypermethylated DMPs were more often located in the OpenSea or the N-Shelf region, and the hypomethylated DMPs were enriched for the S-Shore and N-Shore. At present, we do not know if the enrichment can be attributed to the particular design of the 450K-Illumina Infinium assay or hitherto unknown mechanisms.

The present study provides for the first time evidence of RNA expression changes, not only affecting the chrX but also extending to the autosomes, as well as a widespread DNA hypomethylation and to a lesser extent hypermethylation, in adult women with TS in comparison with female and male controls. We have illustrated that clustering analysis based on either chrX-RNA expression data, autosomal methylation profiles, or the chrX methylation profile clearly distinguishes TS from controls corroborating previous studies^21^. Results show genome-wide changes in RNA expression with the chrX providing the largest amount of DE genes. We point to candidate genes of comorbidity in TS e.g. congenital urinary malformations (*PRKX*), premature ovarian failure (*KDM6A*) and aortic aneurysm (*ZFYVE9* and *TIMP1*). We analyze methylation data from both a single position as well as regional perspective with results showing epigenetic regulation of genes related to embryonic development and morphogenesis in general and TS related comorbidities in particular.

## Methods

### Subjects

Women with karyotypically proven TS and their age-matched 46,XX controls, previously participating in a comprehensive study of the cardiovascular phenotype of TS, were invited to participate^54^. Thirty-five women with 45,X monosomy (200 metaphases) and 24 of the original controls were included. To account for differences in samples size, an additional nine healthy age-match controls were recruited (mean age ± SD: TS: 45.0 ± 10.3; TS = 33 and 46,XX: 42.6 ± 12.3; n = 33). Thirty-three age-matched healthy male controls (43.9 ± 8.63; n = 32 ; oneway anova = 0.7) from a study of the Klinefelter syndrome phenotype (Clinical trial NCT00999310) served as male controls. All participants underwent DNA-methylation profiling, and a subset of 12 women with TS (median age [range]: 37.9 [28;46]), 13 46,XX controls (34.4 [22;45]) and 12 46,XY controls (36.0 [22;47]; Kruskal-Wallis P-value = 0.8) was subjected to gene expression profiling.

Informed written consent was obtained. The ethical committee of The Central Denmark Region approved the study, and all clinical investigation was conducted according to the principles expressed in the Declaration of Helsinki.

### Sample preparation

#### 450K-Illumina Infinium assay

EDTA-treated peripheral blood samples were stored immediately until use at −80 °C. Genomic DNA was extracted using QIAmp Mini Kit (Qiagen, Germany). For each sample, 1 μg of genomic DNA was bisulfite-converted using Zymo EZ DNA-methylation Kit. The methylation level was measured using the Infinium HumanMethylation450 Beadchip Kit (Illumina, Inc.) at Aros Applied Biotechnology A/S.

#### DNA-methylation data preprocessing

Data were analyzed using the R package *minfi*. Detection p-values were calculated to identify failed positions with a p-value cut-off > 0.01. According to these criteria probes were removed if they failed in more than 20% of the samples (n = 361). Individual positions were removed at the specified cut-off. No samples had a proportion of failed probes exceeding 1% or a median intensity below 11. Raw data was normalized implementing the preprocessing defaults of Genome Studio with background normalization and control normalization. Subsequently, we applied subset-quantile-within-array-normalization correcting for technical differences between Infinium type I and II assay design, allowing both within-array and between-sample normalization. Cross-reactive probes (n = 29,541), probes with SNPs documented in the C or G of the target (n = 18,286), and probes on the sex chromosomes (n = 11,352) were excluded, leaving 415,015 probes. Subsequently, methylation values where calculated as M-values (logit[beta]) (Equation (1)) as recommended by *Du et al.*^55^ and as to allow the use of a statistical model adjusting for cell-count.


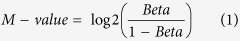


Multidimensional scaling plots were evaluated to identify clusters of samples behaving differently than expected. Two women with 45,X monosomy (ID 32 and 28) were excluded as they were outliers (Supplemental Fig. 14A), and due to the fact, that they were identical twins, leaving three clusters representing the karyotypes 46,XY; 46,XX; and 45,X (Supplemental Fig. 14B). In addition, one outlier male control was excluded (Supplemental Fig. 14C). Finally, probes were annotated to the human genome version 19 using the enhanced Illumina annotation developed by Price *et al*.^23^.

#### Estimate differential cell counts

To account for differences in cell composition *Minfi’s* estimateCellCounts was used returning the relative proportions of CD4+ and CD8+ T-cells, natural killer cells, monocytes, granulocytes, and B-cells in each sample^56^.

#### Identifying differentially methylated positions (DMPs)

To identify positions where methylation is associated with the karyotype, we fitted a linear model, which utilizes a generalized least squares model and an F-test (lmFit of R-package *Limma*), allowing for missing values. The sample variances were squeezed by computing empirical Bayes posterior means. A Bonferroni adjusted family-wise error rate (FWER) below 0.05 was considered significant. The model was applied with adjustment for the estimated relative cell proportions (CD4+ and CD8+ T-cells, natural killer cells, monocytes, granulocytes, and B-cells) as well as for age. Since small changes in M-values might be of spurious biological significance, we added a delta M-value-threshold excluding all DMPs with an absolute delta-M-value ≤ 1. Hierarchical cluster analysis was done using *pvclust*^57^. Pvclust provides approximately unbiased p-value (au) based on multi-scale bootstrap resampling (bootstraps = 1,000) as well as a p-value computed by normal bootstrap resampling (bp). Approximately unbiased p-values and bp p-values are between 0 and 100. Clusters with au larger than 95% are strongly supported by data. Chromosomal and gene-centric-region enrichment analysis was done applying hypergeometric testing to the TS vs. 46,XX comparison in order to identify if any chromosome or gene centric region was enriched for methylation changes.

#### Repetitive elements

We adapted to annotation from Price *et al*. defining repetitive elements as non-cross reacting probes with the entire probe (50 base pairs) in repetitive DNA^23^ allowing assessment of the methylation level at autosomal repeats comparing TS with 46,XX control. We clustered probes if on the same chromosome and separated them by less than 500 base pairs defining in total 1404 regions in addition to the 19,468 Illumina probes hybridizing to repetitive elements. Furthermore, we assessed if the number of DMPs in repetitive elements were more than expected per chance.

#### Identifying differentially methylated regions (DMRs)

Adjacent site clustering was used to identify DMRs (R-package *Aclust*)^58^. Aclust clusters neighboring CpG sites according to a correlation-based distance measure. For testing, Aclust assumes, that the exposure equally affects all CpG sites using generalized estimating equations adapting the R-package, *geepack.* The method was applied on M-values using Pearson’s coefficient of correlation with a correlation-based distance measure of 0.25. Furthermore, CpG sites were limited from clustering if more than 1000 base pairs apart with an absolute Delta-M-value > 0.5. A FWER < 0.05 was considered significant.

#### Functional annotation based on DNA-methylation data

DMPs were annotated to the human genome version 19. DMPsm common to both the TS vs 46,XX and TS vs 46,XY adjusted comparison with a FWER < 0.05, and an absolute(delta-M-value) > 0.5 were considered the most reliable. All genes associated with these DMPs were entered into The Database for Annotation, Visualization and Integrated Discovery (DAVID, http://www.nature.com/nprot/journal/v4/n1/suppinfo/nprot.2008.211_S1.html). The database performs a biological module-centric analysis viewing functionally related genes together as a unit in order to identify the most overrepresented biological terms associated with a given gene list. The DAVID functional annotation clustering measures the relationship among the annotation terms on the basis of the degree of their co-association with genes within the entered gene list to cluster into functional annotation groups. From the gene-centric modular analysis, terms with a Fisher’s exact p-value < 0.05 are reported.

With respect to DMRs the chromosome location as well as start and end position of each were annotated using the Genomic Regions Enrichment of Annotations Tool (GREAT, http://www.nature.com/nbt/journal/v28/n5/abs/nbt.1630.html#supplementary-information). DMRs were mapped to genes, assigned a regulatory domain extended 10 kb proximal and 3 kb downstream of the TSS (transcription start site) with a distal extension of 150 kb, We included the Curated Regulatory Domains option (domains where experimental evidence demonstrates that a gene is directly regulated by an element that falls outside of its putative regulatory domain). Significant GO-terms were extracted.

#### X-chromosome analysis with respect to DNA-methylation

Quality control and normalization were done as for the autosomes. In addition, cross-reactive probes (n = 1201), probes containing SNPs (n = 1078), and known male hypermethylated genes (of the MAGE, GAGE and LAGE gene family) were excluded (n = 193), leaving 8,757 probes to map uniquely to the X-chromosome (chrX). X inactivation of the one X chromosome in normal females leads to a combined X chromosomal methylation pattern which cannot be compared with the one active X in 45,X. We compared the active chrX of TS with the active chrX of normal males applying the aforementioned statistical models to assess DMPs and DMRs, respectively. Cotton *et al*.’s categorization of genes that escape from chrX inactivation (XCI) was applied to classify genes into escape or inactivated genes^59^.

#### RNA-seq sample preparation

Blood samples were drawn using RNApax gene tubes and placed 2 hours at room temperature, sequentially stored overnight at −21 degrees before storage at −80 degrees.

#### RNA-seq library construction and sequencing

Whole transcriptome, strand-specific RNA-Seq libraries facilitating multiplexed paired-end sequencing were prepared from total-RNA using the Ribo-Zero Globin Gold technology (Epicentre, an Illumina company) for depletion of rRNA and globin mRNA followed by library preparation using the ScriptSeq technology (Epicentre, an Illumina company). Depletion and library preparation were automated on a Sciclone NGS (Caliper, Perkin Elmer) liquid handling robot. The total-RNA (1.7 μg per sample) was subjected to Baseline-ZERO DNase prior to depletion. Total-RNA was purified using Agencourt RNAClean XP Beads before and after DNase treatment, followed by on-chip electrophoresis on a LabChip GX (Caliper, Perkin Elmer) and by UV measurements on a NanoQuant (Tecan). Cytoplasmic and mitochondrial rRNA as well as globin mRNA were removed from 400 ng DNase treated total RNA using the Ribo-Zero Globin Gold Kit (Human/Mouse/Rat, Epicentre, an Illumina company) following the manufacturer’s instructions, and the quality of the depleted RNA was estimated on a LabChip GX (Caliper, Perkin Elmer). Synthesis of directional, paired-end and indexed RNA-Seq libraries were conducted using the ScriptSeq v2 kit (Epicentre, an Illumina company) following the recommended procedure, and the qualities of the RNA-Seq libraries were estimated by on-chip electrophoresis (HS Chip, LabChip GX, Caliper, Perkin Elmer) of a 1 μL sample. The DNA concentrations of the libraries were estimated using the KAPA Library Quantification Kit (Kapa Biosystems). The KAPA qPCR reactions were prepared on a Zephyr NGS liquid handling robot (Caliper, Perkin Elmer). The RNA-Seq libraries were multiplexed paired-end sequenced on an Illumina HiSeq 2000 (100 + 6 + 100 bp) or on an Illumina NextSeq (75 + 6 + 75 bp).

#### RNAseq analysis

Paired de-multiplexed fastq files were generated using CASAVA software (Illumina) and initial quality control was performed using FastQC. Adapter trimming was conducted using the GATK ReadAdaptorTrimmer tool followed by mapping to the Human genome (hg19) using Bowtie and then further analyzed using Tophat, and Cufflinks. HTSeq-count (union method) was applied to produce raw counts which were then submitted for analysis in R using edgeR. All non-informative features were removed (n = 5). Filtering was done by removing features with less than one counts per million (cpm) in 12 samples removing 32,458 features, leaving 10,756 for downstream analysis^60^. A generalized linear model was fitted yielding an overall p-value. Secondly, p-values and log fold changes were retrieved from the individual comparisons of TS vs. 46,XX, TS vs. 46,XY, and 46,XX vs 46,XY. Genes mapping to the autosomes were retrieved yielding a matrix of 10,174 genes. MDSplots were used to explore the count tables and to assess if samples clustered according to karyotype (Fig. 1B,D).

Genes from the comparison TS vs. 46,XX were submitted to DAVID for functional annotation pathway analysis using the 10,174 autosomal genes. Categories with a Fisher’s exact test < 0.05 were retrieved.

In order to address the hypothesis that loss of sex-chromosome material manifests itself in differential expression of X-linked genes, crude p-values and log fold changes were retrieved for genes mapping to the sex chromosomes and FDR p-values calculated using Benjamini-Hochberg’s equation.

### X-Y homologs outside the PAR region

Information on X-Y homologs was adapted from Bellott *et al*. Nature 2014 and HGNC gene symbol and ensemble gene id were retrieved, if known. In order to take differing gene length into account Fragments Per Kilobase of Exon values (FPKM) of the X-Y pairs were calculated, then summed, and differential expression was re-assessed with Kruskal-Wallis test and Mann-Whitney U-test.

### Genes of the Pseudo-autosomal Region

Based on annotations from gencode, we generated a list of pseudo-autosomal genes located on the chrX. Using samtools, we counted the primary reads originating from each exon in a strand-specific manor.

Filtering was done by removing features with less than one cpm in 12 samples and thereby removing 51 tags leaving 26 tags. A generalized linear model was fitted yielding an overall p-value. Secondly, p-values and log fold changes were retrieved from the individual comparisons of TS vs. 46,XX, TS vs. 46,XY, and 46,XX vs 46,XY. An absolute log fold change ≥ 0.3 (corresponding to a fold change ≥ 1.2) in conjunction with an overall and individual FDR < 0.05 was considered significant.

In addition to the annotation by Bellott *et al*. we adapted Muellers *et al*. annotation on multi-copy, single-copy, and ampliconic genes^28^.

### Differential exon usage of the X-chromosome

DEXSeq was used to evaluate differential exon usage and results from the female contrast returned. Exon-usage fold changes were calculated by fitting for each gene, a generalized linear mode from the joint data of all its exons. The autosomes and chrX were considered as two independent hypotheses. A FDR < 0.05 was considered significant.

### Analysis software

Statistical computations were performed using R 3.1.0 (R Foundation for Statistical Computing, Vienna, Austria) with Bioconductor 3.0. Methylation was assessed using the minfi, Aclust^58^, as well as Limma package, and RNA-seq data using edgeR. Differential exon usage was assessed and plotted using DEXSeq. Graphics were made using the basic R functions, ggbio, Gviz, DEseq, DEXSeq and ggplot2.

### Subject codes

DNA-methylation, Sex chromosome aneuploidy, Turner syndrome, Whole genome gene expression profile, X-chromosome inactivation. ClinicalTrial.gov Identifier: NCT01678261 (https://clinicaltrials.gov/ct2/show/NCT01678261).

## Additional Information

**How to cite this article**: Trolle, C. *et al*. Widespread DNA hypomethylation and differential gene expression in Turner syndrome. *Sci. Rep.* **6**, 34220; doi: 10.1038/srep34220 (2016).

## Supplementary Material

Supplementary Information

## Figures and Tables

**Table 1:** Characterization of the four X-chromosomal escape genes corresponding to differentially methylated positions with a FWER < 0.05.

HGNC symbol	Function	Diseases
USP9X	Member of the peptidase C19 family. Encodes a protein that is similar to ubiquitin-specific proteases. Known to escape X-inactivation. Mutations in this gene have been associated with Turner syndrome. It has been suggested that USP9X mutations cause changes in the neuronal cytoskeleton, which may affect neuronal migration and axonal growth, resulting in intellectual disability. [http://www.omim.org/entry/300072?search=USP9X&highlight=usp9x]	Turner syndrome, X-linked recessive nonsyndromic mental retardation
UBA1	First step in ubiquitin conjugation to mark cellular proteins for degradation. [http://www.omim.org/entry/314370? search=UBA1&highlight=uba1]	X-linked infantile spinal muscular atrophy?
STS	Catalyzes the conversion of sulfated steroid precursors to estrogen	X-linked ichthyosis
KDM6A	Known Y homolog gene. KDM6A encodes a tetratricopeptide repeat (TPR) protein. The encoded protein contains a JmjC-domain and catalyzes the demethylation of tri/dimethylated histone H3. Known escape gene. Importance for reestablishment of pluripotency and germ cell development	Kabuki Syndrome 2
