# Supplementary material for: Widespread DNA hypomethylation and differential gene expression in Turner syndrome
Source: Sci Rep. 2016 Sep 30;6:34220. doi: 10.1038/srep34220 (PMC5043230; doi:10.1038/srep34220)
Supplement: Supplementary Information [file srep34220-s1.pdf]

Widespread DNA hypomethylation and differential gene expression in Turner syndrome

Christian Trolle, MD[a,c]; Morten Muhlig Nielsen, MSc, PhD[c]; Anne Skakkebæk MD, PhD[b]; Philippe Lamy, MSc[c], PhD[c]; Søren Vang, MSc[c] , PhD[c]; Jakob Hedegaard, MSc[c], PhD[c]; Iver Nordentoft, MSc[c], PhD[c]; Torben Falck Ørntoft, MD, PhD, DMsc[c]; Jakob Skou Pedersen. MSc, PhD[c,d]; Claus Højbjerg Gravholt, MD, PhD DMsc[a,c].

[a]*Department of Endocrinology and Internal Medicine and Medical Research Laboratories, Aarhus University Hospital, Aarhus, Denmark;* [b]*Department of Clinical Genetics, Aarhus University Hospital, Aarhus, Denmark;* [c]*Department of Molecular Medicine, Aarhus University Hospital, Aarhus, Denmark.* [d]*Bioinformatics Research Centre, Aarhus University, Aarhus, Denmark.*

Corresponding author: Christian Trolle, MD, Department of Molecular Medicine, Aarhus University Hospital, Brendstrupgårdsvej 21, 8200 Aarhus N, Denmark, Telephone: +45 6133 9269, Telefax: +45 7846 2010, E-mail: **christian.trolle@clin.au.dk**

ClinicalTrial.gov Identifier: NCT01678261 (https://clinicaltrials.gov/ct2/show/NCT01678261)

Supplemental Figures

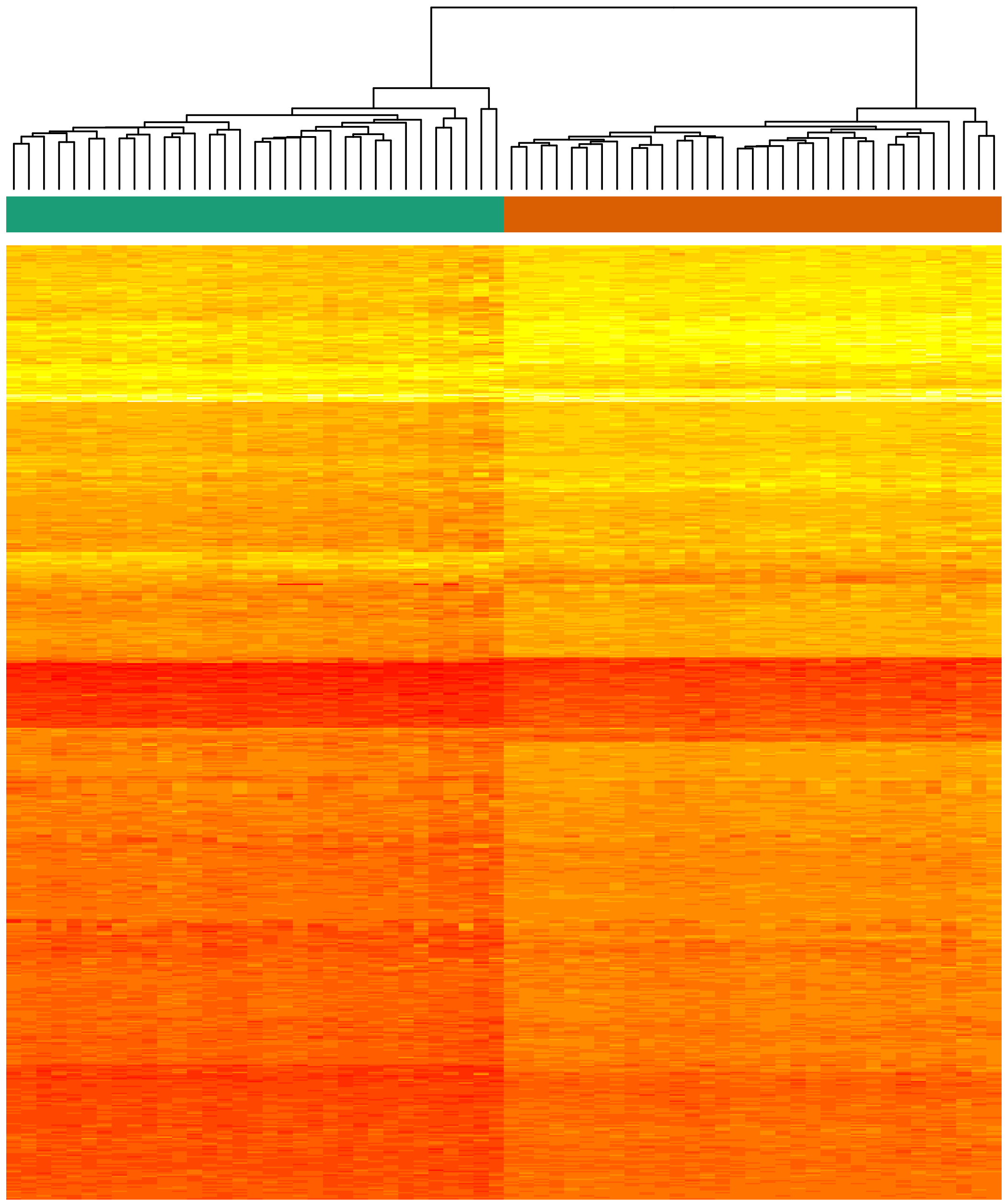

Supplemental Figure 1. Heatmap corresponding to one sample for each column and one DMP for each row using supervised hierchical clustering testing for differences between groups with FWER<0.05 and absolute (Delta-M-value)>1 as cut-off. Red to yellow corresponds to relatively hypomethylated (red) to hypermethylated (yellow). Color bar above corresponds to groups, with green being Turner syndrome and orange Female controls. Dendrogram is displaced at the top.

A.

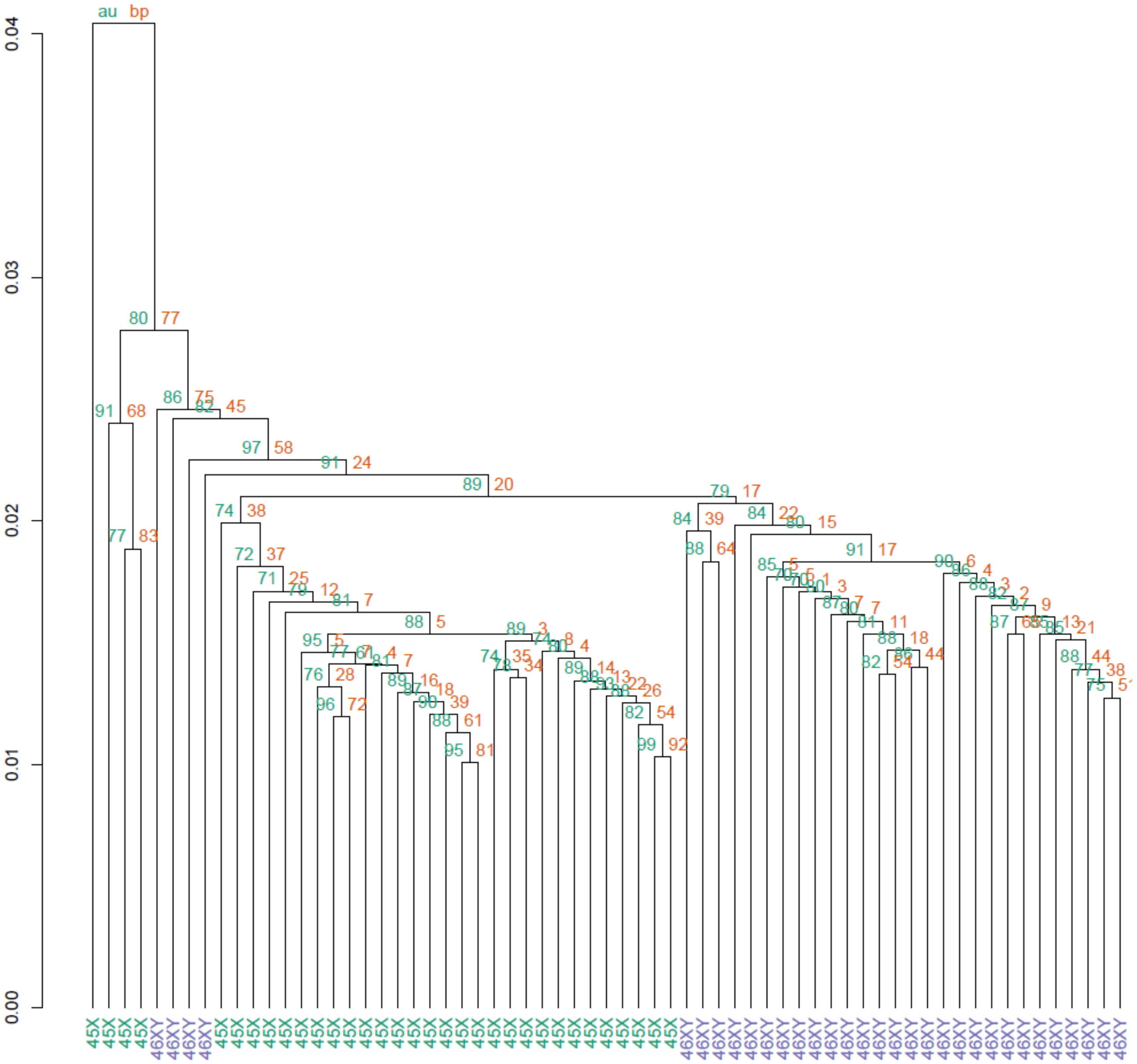

B.

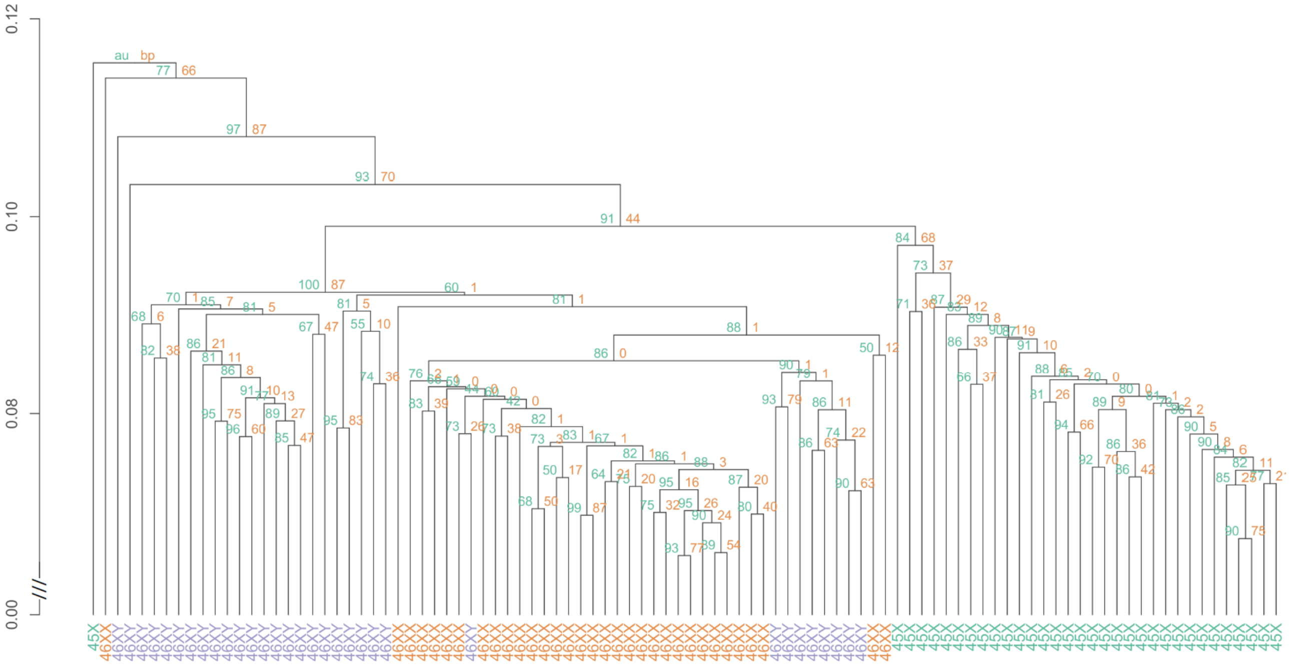

Supplemental Figure 2. Hierarchical cluster analysis to classify individuals according their M-values revealed a distinct Turner syndrome cluster. **Au** is the multiscale bootstrap resampling (bootstraps=1,000) based approximately unbiased p-value (given in red). **Bp** is the normal bootstrap resampling p-value (in green). Both au and bp represents the p-value of a cluster as a value between 0 and 100%. Clusters with an au larger than 95% are strongly supported by data. Labels indicate karyotype (Orange=46,XX; Green=45,X; Purple=46,XY). **A:** X-chromsome analysis, **B:** Autosome analysis.

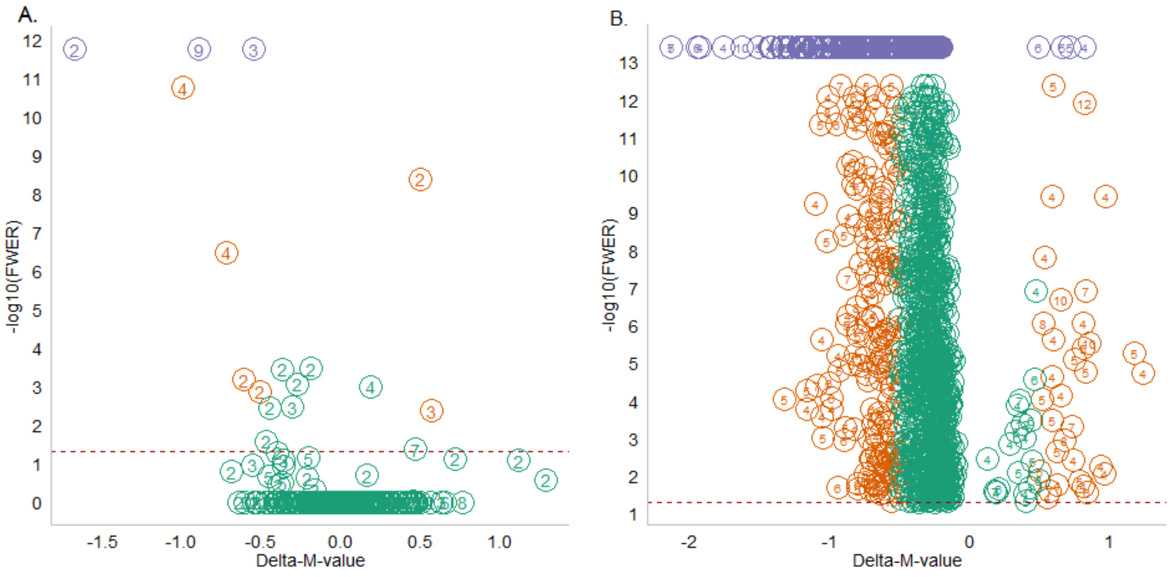

Supplemental Figure 3. Volcanoplot of Aclust derived DMRs. – log10(FWER) against Delta-M-values. 45,X vs. 46,XX) illustrating a predominant 45,X hypomethylation. **A.** X-chromosome data based on the 45,X vs. 46,XY comparison. **B.** Autosome data based on the 45,X vs. 46,XX comparison. Orange: absolute (delta-M-value)>0.5. Purple: FWER below limit of reporting as defined by Aclust. Digits: Number of CpGs in each DMR. Dashed red line: FWER=0.05.

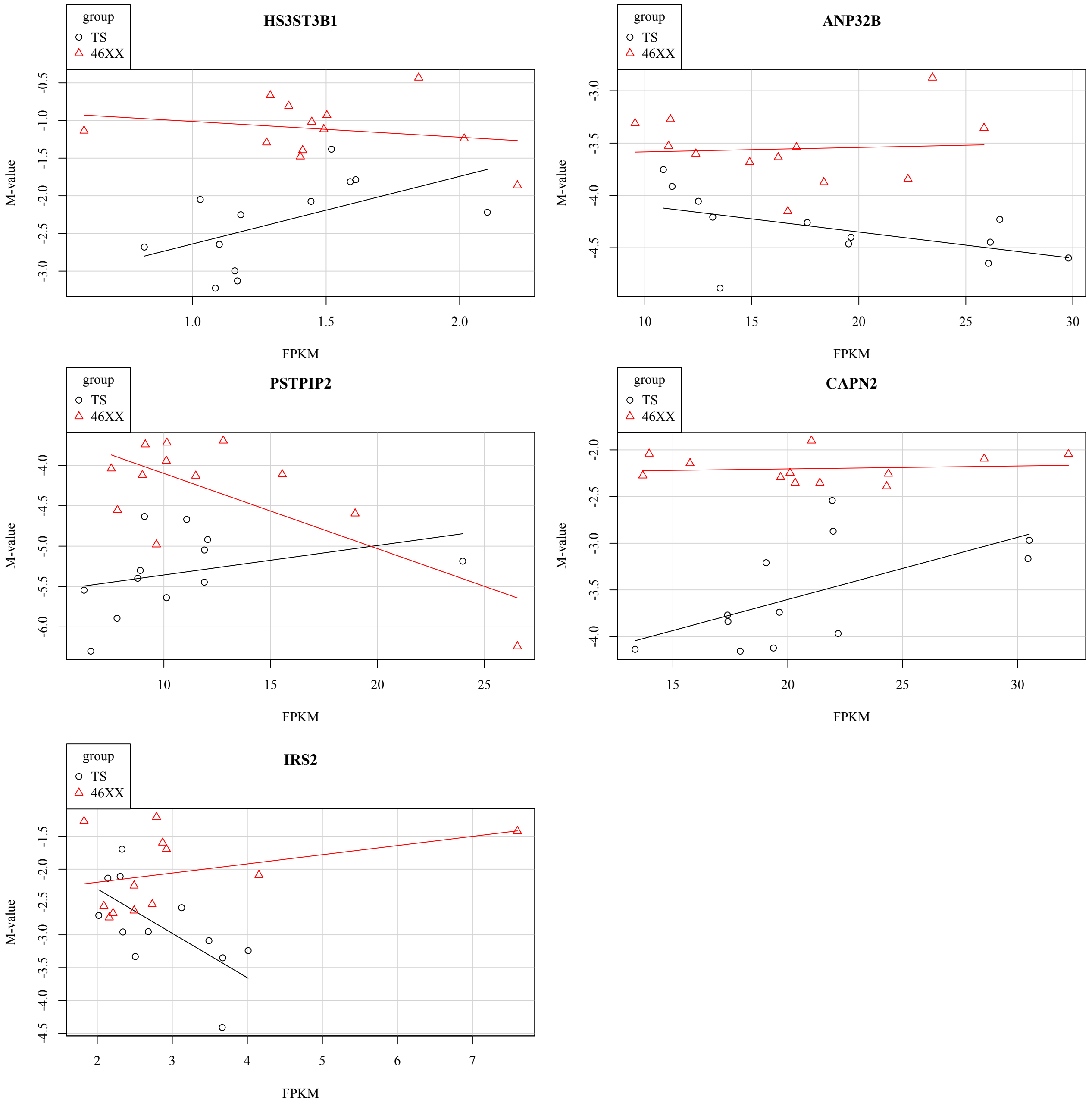

Supplemental Figure 4. DNA-methylation (M-value) plotted against expression (FPKM) corresponding to the five autosomal genes showing a significant correlation in the Turner syndrome group while also being differentially methylated when comparing Turner syndrome and female controls.

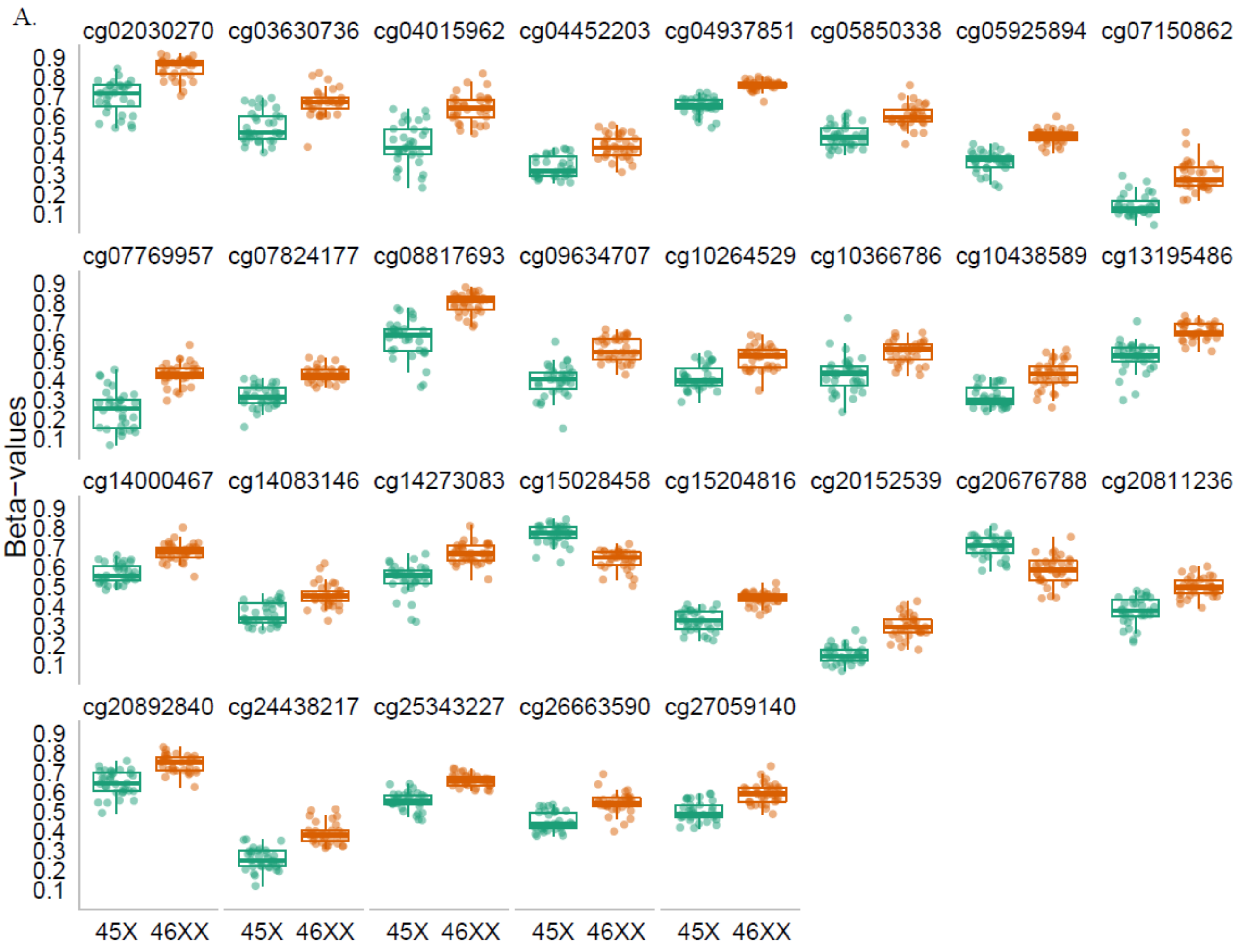
A.
cg02030270
cg03630736
cg04015962
cg04452203
cg04937851
cg05850338
cg05925894
cg07150862
cg07769957
cg07824177
cg08817693
cg09634707
cg10264529
cg10366786
cg10438589
cg13195486
cg14000467
cg14083146
cg14273083
cg15028458
cg15204816
cg20152539
cg20676788
cg20811236
cg20892840
cg24438217
cg25343227
cg26663590
cg27059140
Beta−values
0.9
0.8
0.7
0.6
0.5
0.4
0.3
0.2
0.1
45X
46XX

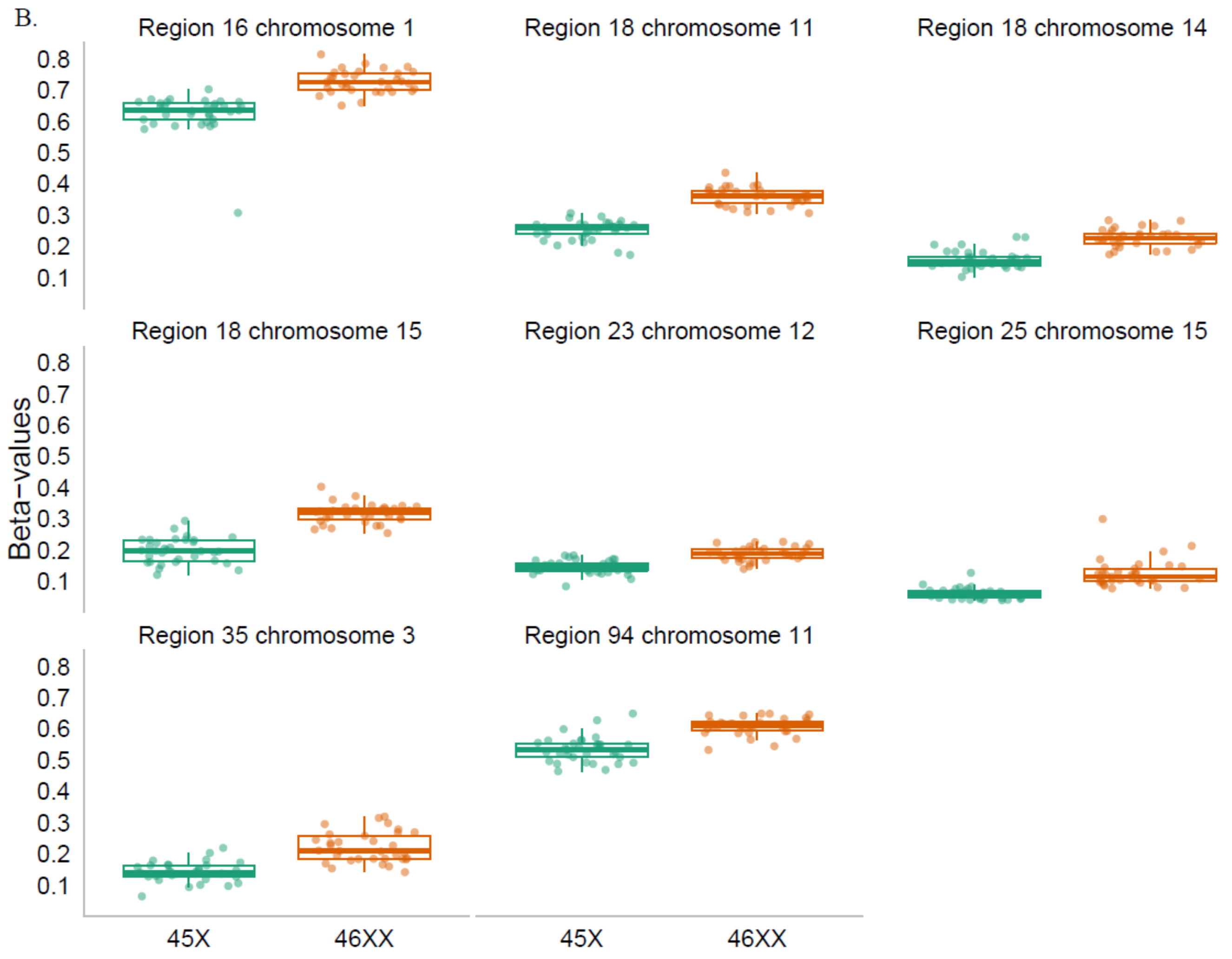

Supplemental Figure 5. A. Beta-values corresponding to DMPs with a FWER<0.05, an absolute (Delta-Beta)>0.1, and mapping to repetitive elements. 27 were hypomethylated and 2 hypermethylated in Turner syndrome compared to 46,XX controls. **B.** Beta-values corresponding to differentially methylated regions with a FWER<0.05 and an absolute (Delta-Beta)>0.1.

A.

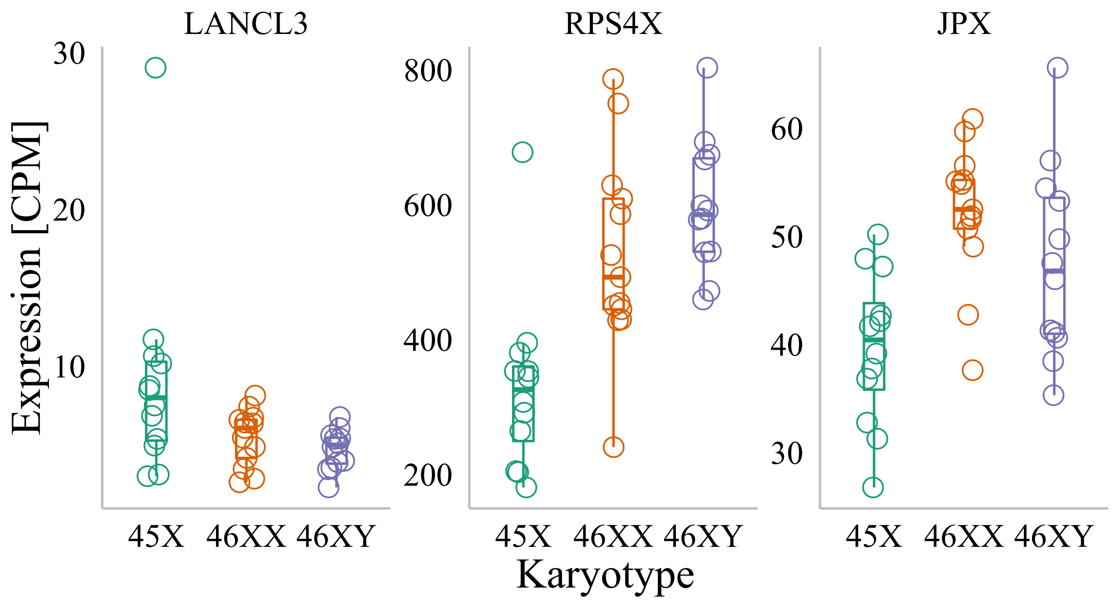

Supplemental Figure 6. Plot of expression values [Counts per million (CPM)] of "Escape" genes differentially expressed in both Turner syndrome comparisons with RPS4X and JPX showing the expected lower expression in Turner syndrome. Contra-intuitively, LANCL3 showed a slight increase.

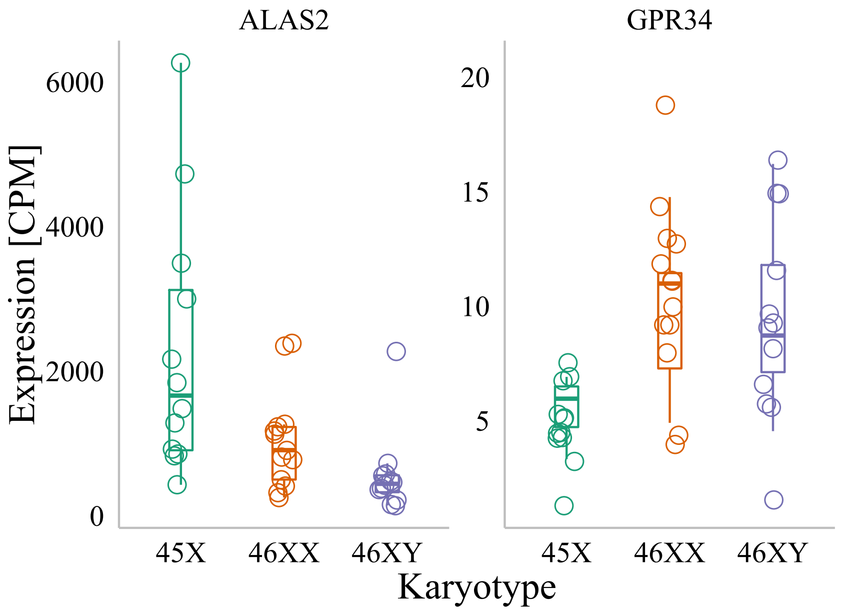

Supplemental Figure 7. Plot of expression values [Counts per million (CPM)] of "Inactivated" genes differentially expressed in both Turner syndrome comparisons.

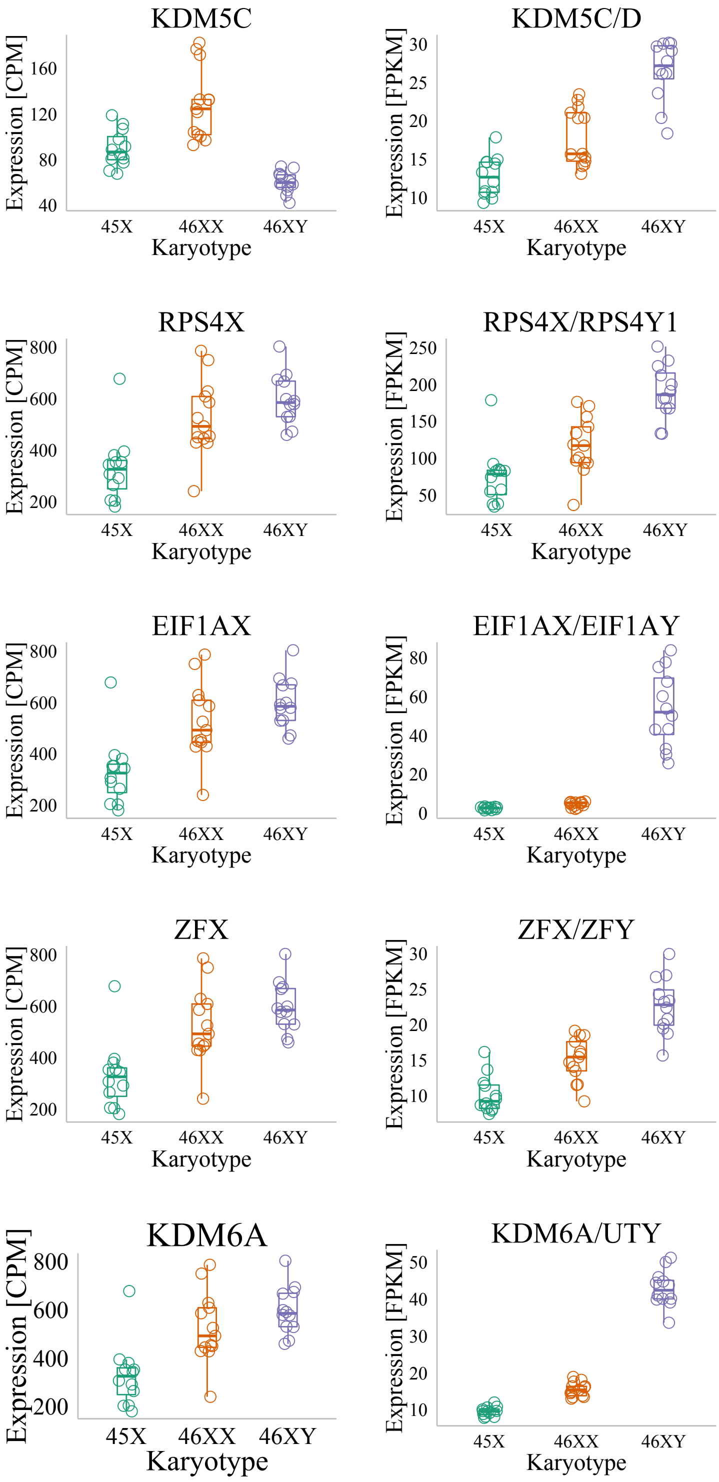

Supplemental Figure 8. Expression of X-Y gene pairs with overall and individual FDR<0.05. Plots illustrate an increased 46,XY expression level when taking into account the Y-copy with no change in the relation between TS and 46,XX. **Left:** Counts per million (CPM) values according to karyotype (FDR<0.05). **Right:** FPKM values summed for X-Y pairs.

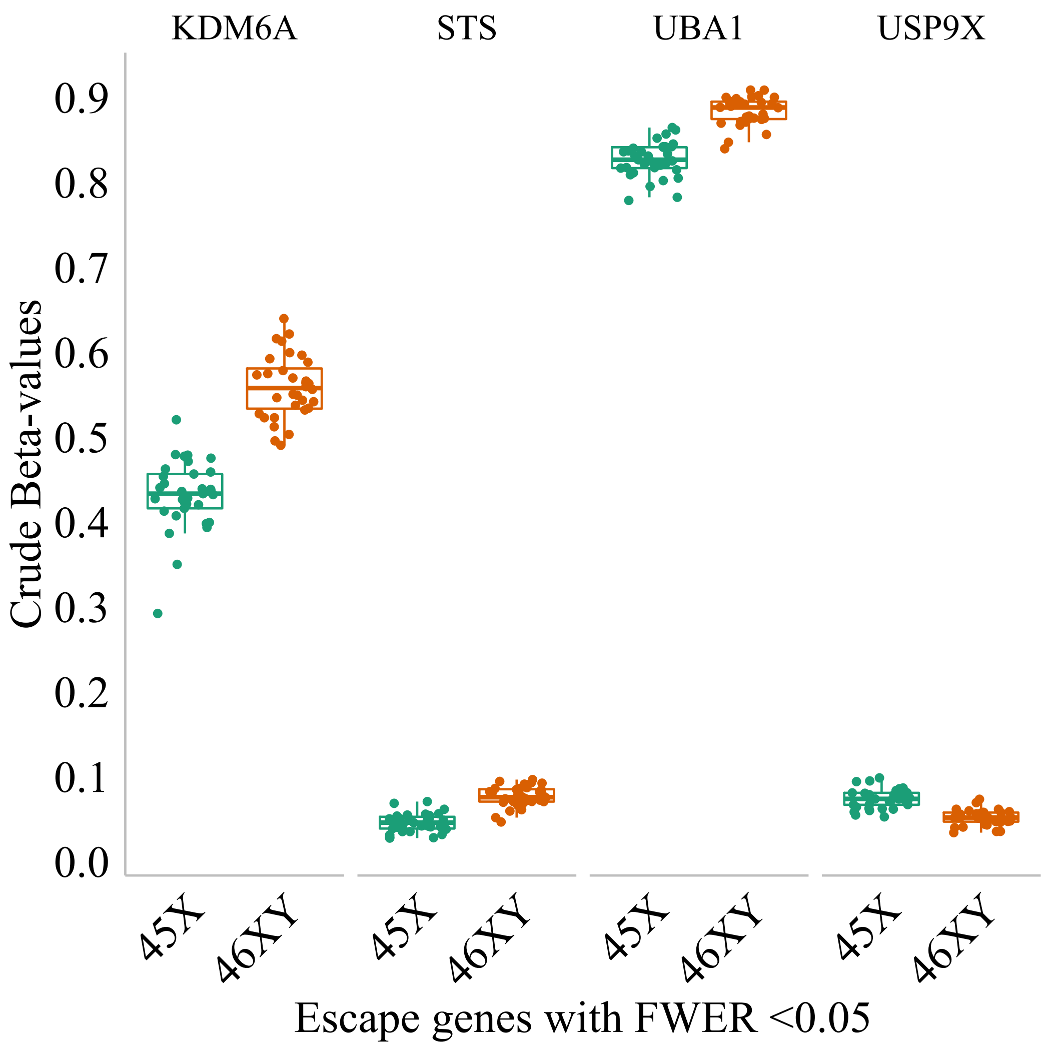

Supplemental Figure 9. Boxplot of crude beta-values of differentially methylated positions with a FWER<0.05 annotated to escape genes. The two most hypermethylated DMPs, KDM6A and UBA1 were located within the gene body and 3'UTR. STS and USP9X were within the proximal promotor.

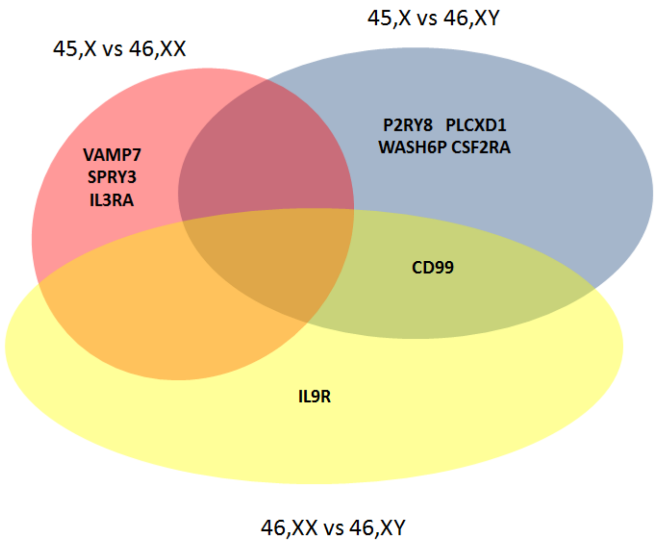

Supplemental Figure 10. Venn diagram listing differentially expressed genes of the pseudo-autosomal region with an overall FDR<0.05, individual FDR<0.05, and an absolute log fold change≥0.3.

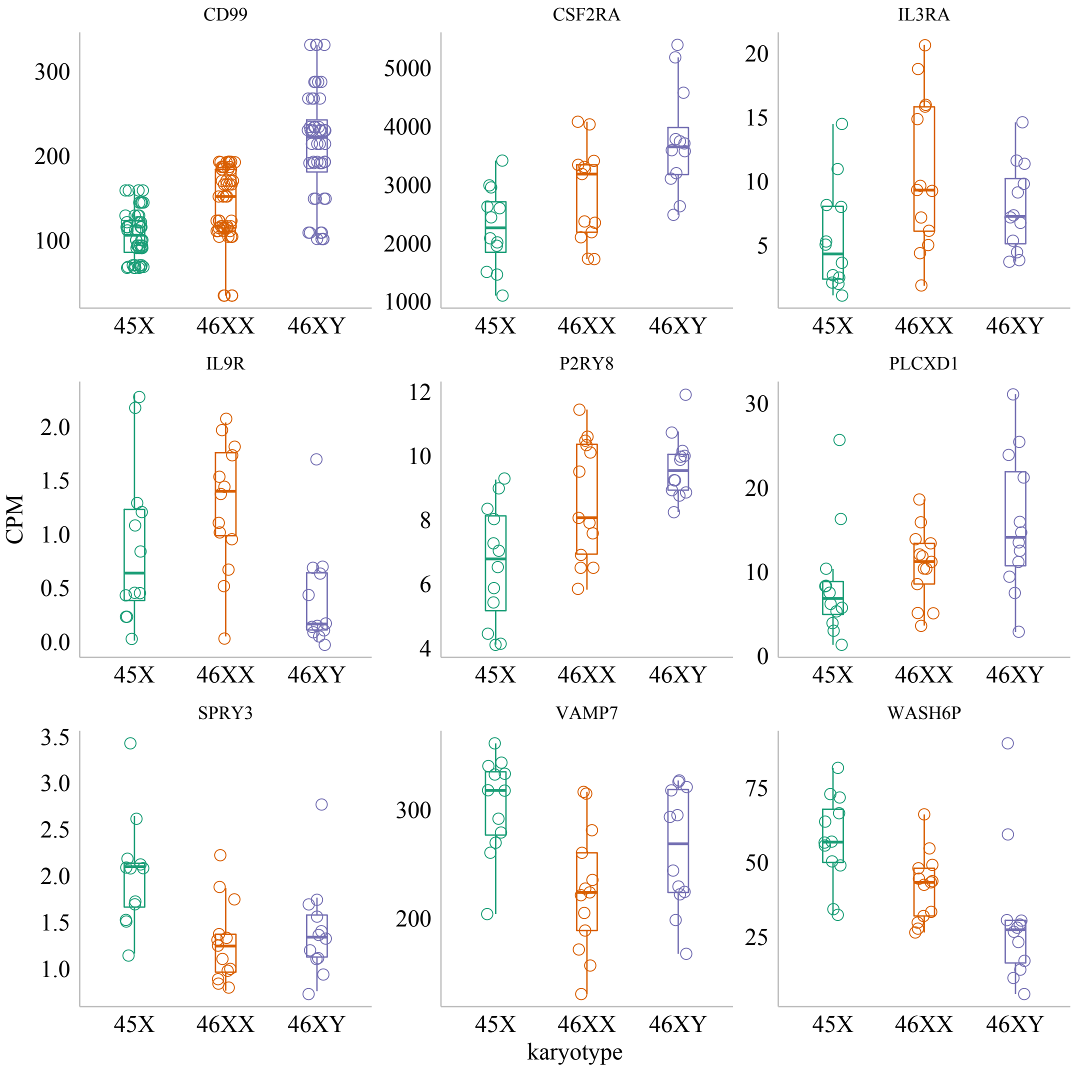

Supplemental Figure 11. Dotplots with overlayed boxplots illustrating the expression of differentially expressed genes of the pseudo-autosomal region with an overall FDR<0.05, individual FDR<0.05, and an absolute log fold change≥0.3.

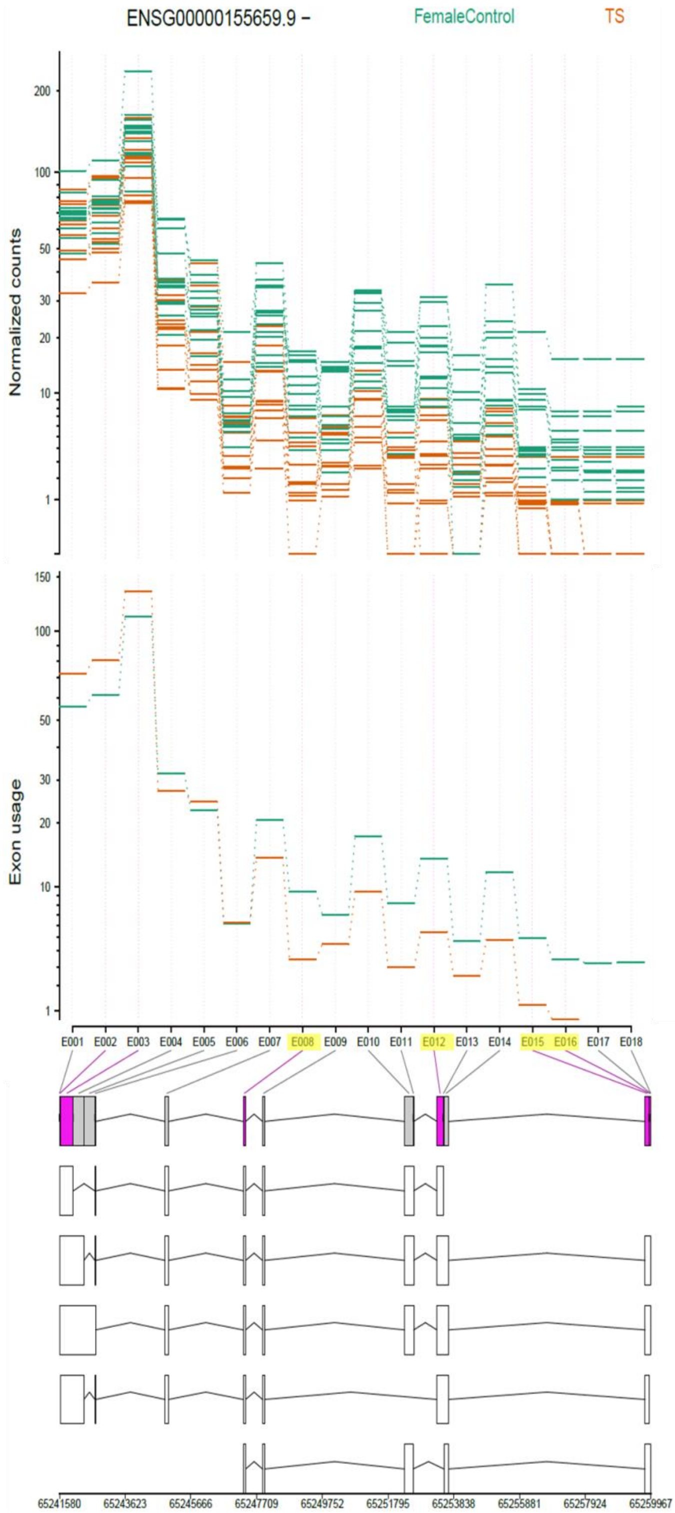

Supplemental Figure 12. Differential exon-usage shown for the *VSIG4* gene. **Top**: Normalized expression estimates. **Middle**: Normalized expression estimates per sample, per exon illustrating that the differential exon-usage is not merely a consequence of the overall down or up-regulation of

the *VSIG4* gene. **Bottom**: Transcript perspective. X-axis is Exons. Highlighted in yellow are exons with FDR<0.05.

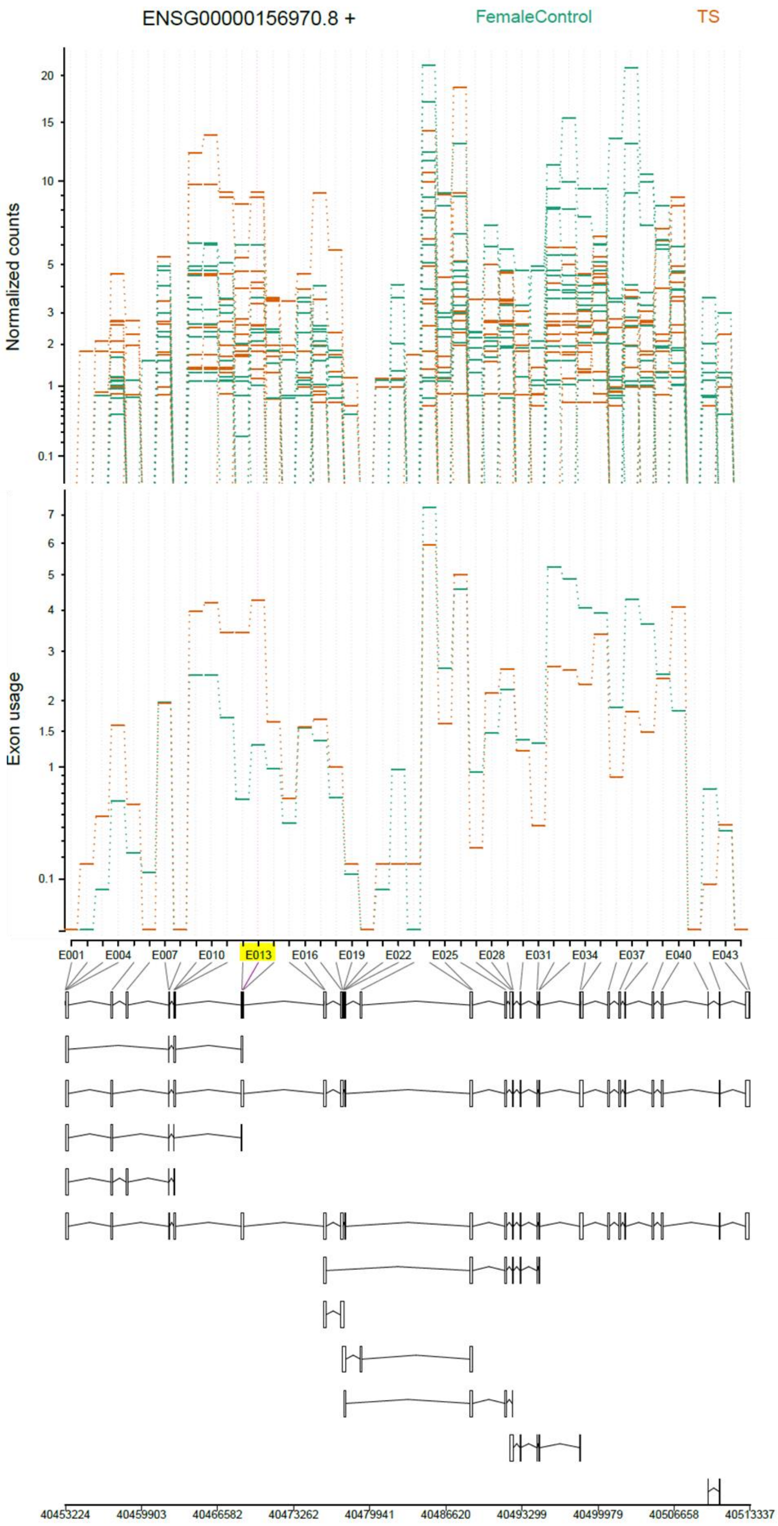

Supplemental Figure 13. Differential exon-usage shown for the BUB1B gene. **Top**: Normalized expression estimates. **Middle**: Normalized expression estimates per sample, per exon illustrating that the differential exon-usage is not merely a consequence of the overall down or up-regulation of

the BUB1B gene. **Bottom**: Transcript perspective. X-axis is Exons. Highlighted in yellow are exons with FDR<0.05.

A.

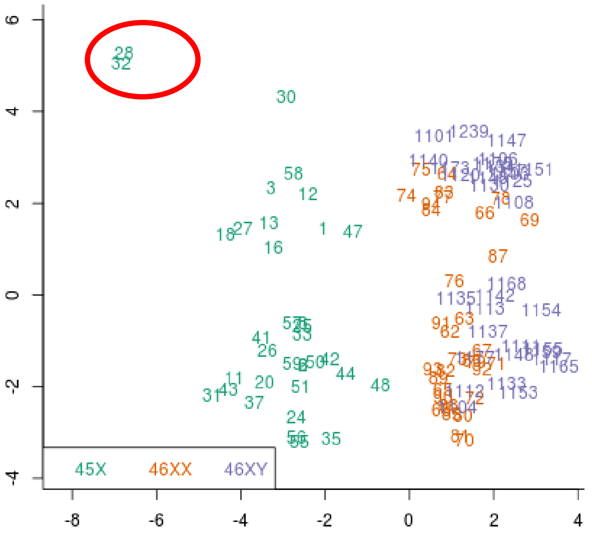

B.

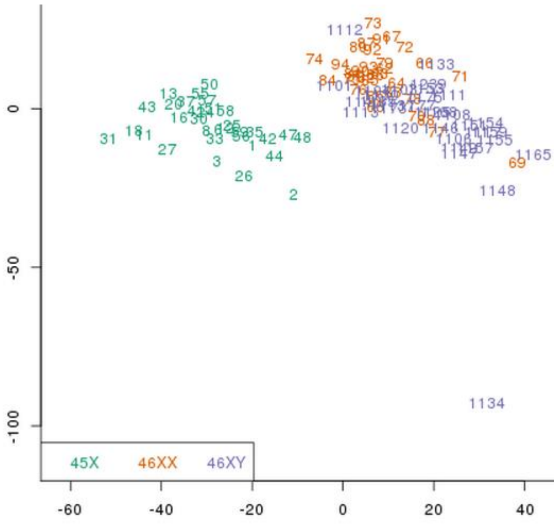

C.

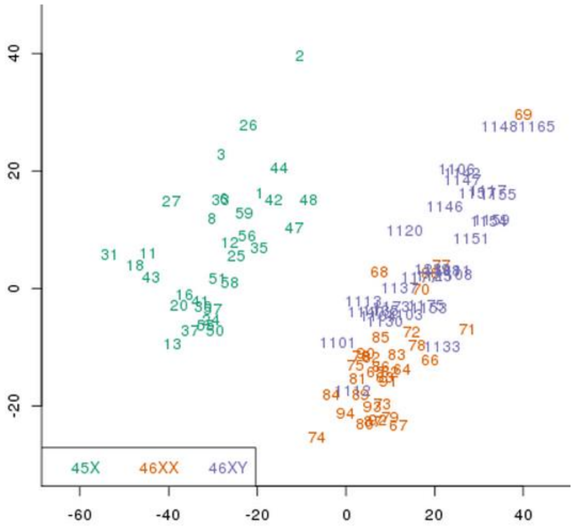

Supplemental Figure 14. Multi-dimensional scaling plot, based on M-values derived from the autosomes, showing a 2-d projection of the Euclidean distances calculated between samples using the 5000 most variable CpG positions. Blue=46,XY males, Orange=46,XX women, Green=45,X monosomy. A. Plot including all samples displaying the two outliers (sample 28 and 32) marked by the red circle. B. Same plot after exclusion of sample 28 and 32. C. Same plot after exclusion of sample 1134.

Supplemental Tables

Gene	Chromosome	Function	Disease	Link
ZNF532	18	Zinc finger protein 532		
IPCEF1	6	Exchange factors 1		
KLHL42	12	Kelch-like family member 42		
TRIM2	4	TRIM2 functions as an E3 ubiquitin ligase that directs proteasome-mediated degradation of target proteins	Charcot-Marie-Tooth disease type 2R	OMIM
DOCK7	1	Guanine nucleotide exchange factor for RAC1 and RAC3, plays a role in neurogenesis. Thought to play a critical role in normal distribution and function of dermal and follicular melanocytes	Epileptic encephalopathy, early infantile	OMIM
CNR1	6	Encode cadherin-like cell surface proteins that are expressed in neurons and are present at synaptic junctions		OMIM
MT2A	16	Metallothionein II processed pseudogene		OMIM
DSC2	18	The cadherins are a superfamily of calcium-dependent glycoproteins that are cell adhesion molecules	Arrhythmogenic right ventricular dysplasia	OMIM
PHLDA1	12	T-cell death-associated gene 51 in mice models is induced by homocysteine, promotes detachment-mediated apoptosis, and contributes to the development of atherosclerosis in hyperhomocysteinemia.	Atherosclerosis	OMIM
IGFBP3	7	Major carrying protein for IGF1 and IGF2 modulator of IGF bioactivity and as a direct growth inhibitor in the extravascular tissue compartment		OMIM
PFKM	12	Encodes the muscle isoform of phosphofructokinase (PFK). Catalyzes the irreversible conversion of fructose-6-phosphate to fructose-1,6-bisphosphate and is a key regulatory enzyme in glycolysis	Glycogen storage disease type VII	OMIM
SCOC	4	Short coiled-coil protein		
ZFYVE9	1	SMAD anchor for receptor activation functions to recruit SMAD2 to the transforming growth factor-beta (TGFB) receptor by controlling the subcellular localization of SMAD2 and by interacting with the TGF-β receptor complex		OMIM
CD200R1	3	Myeloid cell-specific surface glycoprotein that interacts with OX2		OMIM
CXCL5	4	Epithelial cell-derived neutrophil-activating peptide ENA78 is an inflammatory chemokine that is produced concomitantly with interleukin-8 (IL8) in response to stimulation with either interleukin-1 (IL1B) or tumor necrosis factor-alpha (TNF-α)		OMIM
RNASE2	14	Eosinophil-derived neurotoxin		OMIM
SOCS6	18	Suppressor of cytokine signaling 6	CML? Human erythroleukemia?	OMIM
S100z	5	Calcium-binding protein, zeta contains two calcium-binding EF-hands and exhibit cell-type specific expression patterns.		OMIM

SLC22A5	5	Organic cation transporter 2 is a physiologically important, high affinity carnitine transporter that shows significant sodium ion dependence	Inflammatory bowel disease. Carnitine deficiency, systemic primary	OMIM
C5orf42	5	Chromosome 5 open reading frame 42	Joubert syndrome 17, Orofaciodigital syndrome VI	OMIM
GSTM2	1	This enzyme catalyzes the reaction of glutathione with a wide variety of organic compounds to form thio-ethers, a reaction that is sometimes a first step in a detoxification process leading to mercapturic acid formation		OMIM
AC009299.3	2			
HCG11	6	HLA complex group 11 (non-protein coding)		LINK
CHCHD10	22	CHCHD10 is a relatively small protein of the mitochondrial intermembrane space that is enriched at cristae junctions. It is predicted to be involved in oxidative phosphorylation or in maintenance of cristae morphology	Amyotrophic Lateral Sclerosis-2, Spinal Muscular Atrophy, Jokela Type, Autosomal Dominant Isolated Mitochondrial Myopathy	OMIM
OVCH1-AS1	12	OVCH1 antisense RNA 1		LINK
ADGRA3	4	G protein-coupled receptor 125		OMIM

Supplemental Table 1. Descriptives on the 26 differential expressed autosomal genes common to both Turner syndrome comparisons.

Gene	CGI position	Chromosome	rho	P-Value
HS3ST3B1	Orphan	17	0.59	0.049
ANP32B	Orphan	9	-0.64	0.028
PSTPIP2	Orphan	18	0.60	0.043
CAPN2	Orphan	1	0.61	0.040
IRS2	Orphan	13	-0.73	0.010

Supplemental Table 2. Correlation between autosomal gene expression and the autosomal DNA-methylation level at CGIs defined as orphan (within of between characterized transcription units) in Turner syndrome. Only differentially methylated positions (FWER<0.05) from the Turner syndrome versus female controls were included in the analysis.

Gene	XCI status	FC 45X vs 46XX	FC 45X vs 46XY	Function	Disease	Link
LANCL3	Escape	1.67	1.95			
RPS4X	Escape	0.63	0.55	Encodes ribosomal protein S4	XY gonadal dysgenesis patients with somatic features of the Turner syndrome have been found to have deletion of this portion of Yp. However, patients with 46,Xi(Xq) karyotype, i.e., isochromosome Xq, cannot be differentiated phenotypically from 45,X Turner syndrome patients but carry 3 copies of the RPS4X gene	OMIM
ALAS2	Inactivated	2.19	4.03	Catalyzes the first committed step of heme biosynthesis	Hereditary sideroblastic anemia	OMIM
GPR34	Inactivated	0.59	0.62	G protein-coupled receptor		OMIM
JPX	Possibly escaping inactivation	0.76	0.83	Proposed that *XIST* is controlled by 2 parallel RNA switches: TSIX, which represses *XIST* on the active X-chromosome, and JPX, which activates *XIST* on the inactive X-chromosome	Deletion or knockdown of Jpx in female ES cells caused severe abnormalities during ES cell differentiation, including reduced Xist expression, defects in cell outgrowth and attachment, and massive cell death.	OMIM

Supplemental Table 3. Characteristics corresponding to genes differentially expressed when comparing TS to female controls as well as male controls. XCI=The known X-chromosome inactivation status of the gene. FC=Fold change with the value 1 corresponding to no change.

X_Ensembl	HumanXCIEscape	X_hgnc_symbol	Y_Ensembl	Y_Gene	X.Y.Pair	Gene_Ensemble
ENSG00000101825	Escaper	MXRA5	ENSG00000235649	MXRA5Y	Yes	PseudoOnY
**ENSG00000183943**	**Escaper**	**PRKX**	**ENSG00000099725**	**PRKY**	**Yes**	**PRKY**
ENSG00000146938	Escaper	NLGN4X	ENSG00000165246	NLGN4Y	Yes	NLGN4X
ENSG00000101849	Escaper	TBL1X	ENSG00000092377	TBL1Y	Yes	TBL1Y
ENSG00000125363	No Data	AMELX	ENSG00000099721	AMELY	Yes	AMELX
ENSG00000205542	No Data	TMSB4X	ENSG00000154620	TMSB4Y	Yes	TMSB4Y
ENSG00000046651	Escaper	OFD1		OFD1Y	Yes	Missing
ENSG00000169249	No Data	ZRSR2		ZRSR2Y	Yes	Missing
ENSG00000086712	Escaper	TXLNG	ENSG00000131002	TXLNGY	Yes	Missing
**ENSG00000173674**	**Escaper**	***EIF1AX***	**ENSG00000198692**	**EIF1AY**	**Yes**	**EIF1AY**
ENSG00000130741	Escaper	EIF2S3		EIF2S3Y	Yes	Missing
**ENSG00000005889**	**Escaper**	**ZFX**	**ENSG00000067646**	**ZFY**	**Yes**	**ZFY**
ENSG00000124486	Escaper	USP9X	ENSG00000114374	USP9Y	Yes	USP9Y
ENSG00000215301	Escaper	DDX3X	ENSG00000067048	DDX3Y	Yes	DDX3Y
**ENSG00000147050**	**Escaper**	**KDM6A**	**ENSG00000183878**	**UTY**	**Yes**	**UTY**
ENSG00000182872	Nonescaper	RBM10		RBM10Y	Yes	Missing
ENSG00000130985	Escaper	UBA1		UBE1Y	Yes	Missing
ENSG00000068308	Nonescaper	OTUD5		OTUD5Y	Yes	Missing
ENSG00000184205	Nonescaper	TSPYL2		TSPY	Yes	Missing
**ENSG00000126012**	**Escaper**	***KDM5C***	**ENSG00000012817**	**KDM5D**	**Yes**	**KDM5D**
ENSG00000086758	Escaper	HUWE1		HUWE1Y	Yes	Missing
**ENSG00000198034**	**Escaper**	**RPS4X**	**ENSG00000129824**	**RPS4Y1**	**Yes**	**RPS4Y1**
ENSG00000085224	Nonescaper	ATRX		ATRY	Yes	Missing
ENSG00000125676	Nonescaper	THOC2		THOC2Y	Yes	Missing
ENSG00000156531	Escaper	PHF6		PHF6Y	Yes	Missing
ENSG00000156504	Escaper	FAM122B		FAM122BY	Yes	Missing
ENSG00000147274	Nonescaper	RBMX	ENSG00000234414	RBMY1A1	Yes	RBMY1A1
ENSG00000134595	No Data	SOX3	ENSG00000184895	SRY	Yes	SRY
ENSG00000171116	No Data	HSFX1		HSFY	Yes	Missing
ENSG00000029993	Nonescaper	HMGB3		HMGB3Y	Yes	Missing
ENSG00000172534	Escaper	HCFC1		HCFC1Y	Yes	Missing
ENSG00000169057	Nonescaper	MECP2		MECP2Y	Yes	Missing
ENSG00000147403	Nonescaper	RPL10		RPL10Y	Yes	Missing

Supplemental Table 4. Adapted from Bellott *et al* [11]. The pseudo-autosomal region is excluded. Column indicating if the Y-homolog has a known ensemble gene id and official gene symbol has been added as Y_Ensembl and Gene_Ensembl. Rows in bold are genes significant in the 45,X vs. 46,XX comparison.

Term	Count	%	Fisher's Exact	TS comorbidity
Insulin; glucose; polycystic ovary syndrome	3	0.6	4.60E-04	T2D, MS
Disc degeneration, lumbar spine	2	0.4	8.50E-04	Scoliosis
Sciatica	2	0.4	8.50E-04	Scoliosis
Polycystic ovarian syndrome; insulin resistance	2	0.4	8.50E-04	T2D, MS
Intervertebral disc disease	2	0.4	8.50E-04	
Insulin secretion	2	0.4	8.50E-04	T2D, MS
Insulin; blood pressure, arterial	2	0.4	4.90E-03	T2D, MS, HT
Disc degeneration	2	0.4	8.10E-03	Scoliosis
Body mass; birth weight; height	2	0.4	8.10E-03	Obesity, SS
Colon cancer rectal cancer	2	0.4	1.20E-02	Colon cancer
Multiple epiphyseal dysplasia	2	0.4	1.60E-02	Madelung deformity and others
Insulin; obesity	2	0.4	2.10E-02	T2D, MS
Disc degeneration, intervertebral	2	0.4	2.10E-02	Scoliosis
Diabetes, type 2; liver disease	4	0.8	2.40E-02	T2D, ELE
Insulin resistance	3	0.6	2.80E-02	T2D, MS
Insulin	4	0.8	3.30E-02	T2D, MS
Rhinitis	2	0.4	4.70E-02	
Hearing loss, sensorineural non-syndromic	2	0.4	4.70E-02	Impaired Hearing

Supplemental Table 5. Gene-centric modular analysis using DAVID entering autosomal DMPs reaching an FWER p-value <0.05 and absolute (delta-M-value)>0.5. Output from the Genetic Association Database. Last column is comorbidity know to be increased in TS[29], which falls within each category. MS=Metabolic syndrome, T2D=Type 2 diabetes, HT=Hypertension, SS=Short stature, ELE=Elevated liver enzymes, TS=Turner syndrome.

Term	Fisher's	CNR1	CXCL5	RNASE2	ADGRA3	DOCK7	IGFBP3	LRRN3	SOCS6	ZFYVE9	MT2A	PFKM	SLC22A5
Behavior	0.005	■	■	■									
G-protein coupled receptor protein signaling pathway	0.005	■	■		■								
Chemotaxis	0.009		■	■									
Taxis	0.009		■	■									
Regulation of protein amino acid phosphorylation	0.018					■	■						
Locomotory behavior	0.021		■	■									
Negative regulation of signal transduction	0.028						■		■				
Cell surface receptor linked signal transduction	0.029	■	■		■		■			■			
Negative regulation of cell communication	0.032						■		■				
Homeostatic process	0.040										■	■	■
Regulation of growth	0.048						■		■				

Supplemental Table 6. GO-terms from the Functional Pathway Analysis using DAVID achieved by submitting the 33 differentially expressed autosomal genes common to the TS vs. female controls comparison. Only results reaching a Fisher's exact test<0.05 are listed.

GO Biological Process term	Binomial test		Hypergeometric test	
	**FDR**	**FE**	**FDR**	**FE**
Chromosome organization	5.3E-8	2.1	4.8e-2	1.5
Embryonic organ development	8.3e-8	2.3	6.5e-2	1.9
Negative regulation of transcription from RNA polymerase II promoter	8.4e-8	2.1	4.5e-2	1.6
Skeletal system development	9.7e-8	2.3	3.7e-2	1.8
Regionalization	2.3e-7	2.5	4.0e-2	2.0
Embryo development ending in birth or egg hatching	7.7e-7	2.0	3.9e-2	1.6
Chordate embryonic development	1.2e-6	2.0	4.7e-2	1.6
Embryonic skeletal system morphogenesis	3.9e-4	3.4	3.8e-2	2.9
Embryonic skeletal system development	8.0e-4	2.8	3.3e-2	2.6

Supplemental Table 7. Statistical significant gene ontology (GO) terms found using GREAT. Autosomal DMRs with an FWER<0.05 and absolute (delta-M-value)>0.5 were given as input. Association rule: Basal extension: 10kb upstream, 1 kb downstream, 150kb max extension and allowing for curated regulatory domains. FDR=False discovery rate. FE=Fold enrichment.

Chr	Gene	Function	Disease
12	HAL	Histidase is a cytosolic enzyme catalyzing the first reaction in histidine catabolism	Histidinemia
12	ACSS3	Acyl-CoA synthetase short chain family member 3. Plays a role for the incorporation of fatty acids into membranes and signaling molecules and have roles in energy storage and metabolism	
1	STIL	Contains a putative nuclear localization signal and a cysteine-terminal domain similar to the C-terminal domain of TGF-beta. Expression of the STIL gene in human fetal brain and suggested that it may be involved in neuronal cell proliferation.	Microcephaly
22	TUBGCP6	Codes for GCP6 a core component of centrosomes	microcephaly and chorioretinopathy
2	SPAG16	Sperm-associated antigen16. SPAG16 encodes 2 major proteins that associate with the axoneme of sperm tail and the nucleus of postmeiotic germ cells, respectively	
15	BUB1B	Plays a critical role in regulating the spindle-assembly checkpoint	Premature Chromatid Separation Trait and Mosaic Variegated Aneuploidy Syndrome. Colorectal cancer.
4	RGS12	Regulator of G-Protein signaling	
16	SNTB2	Syntrophin beta-2. Abundant in heart and skeletal muscle	

Supplemental Table 8. Autosomal genes showing differential exon-usage. Chr=Chromosome. Gene=HGNC gene symbol.

References

1 Bellott, D. W. *et al.* Mammalian Y chromosomes retain widely expressed dosage-sensitive regulators. *Nature* **508**, 494-499, doi:10.1038/nature13206 (2014).

2 Gravholt, C. H., Juul, S., Naeraa, R. W. & Hansen, J. Morbidity in Turner syndrome. *J Clin.Epidemiol.* **51**, 147-158 (1998).
